# Lung Cancer Cell-Derived Secretome Mediates Paraneoplastic Inflammation and Fibrosis in Kidney in Mice

**DOI:** 10.3390/cancers12123561

**Published:** 2020-11-28

**Authors:** Chi-Chih Hung, Yen-Yi Zhen, Sheng-Wen Niu, Jui-Feng Hsu, Tai-Huang Lee, Hsiang-Hao Chuang, Pei-Hui Wang, Su-Chu Lee, Pi-Chen Lin, Yi-Wen Chiu, Chien-Hsing Wu, Ming-Shyan Huang, Michael Hsiao, Hung-Chun Chen, Chih-Jen Yang

**Affiliations:** 1 Division of Nephrology, Department of Internal Medicine, Kaohsiung Medical University Hospital, Kaohsiung Medical University, Kaohsiung 80708, Taiwan; chichi@cc.kmu.edu.tw (C.-C.H.); R050192@kmu.edu.tw (Y.-Y.Z.); 950138@kmuh.org.tw (S.-W.N.); 700018@kmuh.org.tw (S.-C.L.); Chiuyiwen@kmu.edu.tw (Y.-W.C.); 2 Regenerative Medicine and Cell Therapy Research Center, Kaohsiung Medical University, Kaohsiung 80708, Taiwan; 3 Department of Internal Medicine, Kaohsiung Municipal Ta-Tung Hospital, Kaohsiung Medical University, Kaohsiung 80145, Taiwan; 940360@kmuh.org.tw; 4 Graduate Institute of Medicine, College of Medicine, Kaohsiung Medical University, Kaohsiung 80708, Taiwan; 5 Division of Hematology and Oncology, Department of Internal Medicine, Kaohsiung Medical University Hospital, Kaohsiung Medical University, Kaohsiung 80708, Taiwan; 6 Division of Pulmonary and Critical Care Medicine, Department of Internal Medicine, Kaohsiung Medical University Hospital, Kaohsiung Medical University, Kaohsiung 80708, Taiwan; 1080208@kmuh.org.tw (T.-H.L.); R050397@kmu.edu.tw (H.-H.C.); R920039@kmu.edu.tw (P.-H.W.); 7 Division of Endocrinology and Metabolism, Department of Internal Medicine, Kaohsiung Medical University Hospital, Kaohsiung Medical University, Kaohsiung 80708, Taiwan; pichli@kmu.edu.tw; 8 Division of Nephrology, Department of Internal Medicine, Kaohsiung Chang-Gung Memorial Hospital, Kaohsiung 83301, Taiwan, and College of Medicine, Chang-Gung University, Taoyuan 33303, Taiwan; Vjf3@cgmh.org.tw; 9 Department of Internal Medicine, E-Da Cancer Hospital, School of Medicine, I-Shou University, Kaohsiung 82445, Taiwan; Ed110209@edah.org.tw; 10 Genomics Research Center, Academia Sinica, Taipei 11529, Taiwan; mhsiao@gate.sinica.edu.tw; 11 Department of Biochemistry, College of Medicine, Kaohsiung Medical University, Kaohsiung 80708, Taiwan; 12 Department of Respiratory Therapy, College of Medicine, Kaohsiung Medical University, Kaohsiung 80708, Taiwan

**Keywords:** paraneoplastic syndromes, albuminuria, secretome, renal fibrosis, IL-6, MCP-1, TGF-β

## Abstract

**Simple Summary:**

Paraneoplastic nephrotic syndrome is a complication arising in lung cancer patients. In the present study, we established an LLC1 cell orthotopic xenograft C57BL/6 mice model to translation paraneoplastic nephrotic syndrome (PNS). The pathological aspects of PNS were characterized in TGF-β signaling-engaged renal fibrosis, and renal inflammation with IL-6 expression in kidney. To reveal how the lung cancer cells remotely drive pathogenic progression, secretome derived from LLC1 cells and A549 cells were proteomically profiled. Additionally, the secretome profiling was subjected to diseases and biofunctions assessment by Ingenuity Pathway analysis (IPA). As matter of secretome profiling and IPA prediction, the Fibronectin, C1r, and C1s are potential of nephrotoxicity linked to paraneoplastic effects on glomerular pathogenesis in these lung cancer mice.

**Abstract:**

Kidney failure is a possible but rare complication in lung cancer patients that may be caused by massive tumor lysis or a paraneoplastic effect. Clinical case reports have documented pathological characteristics of paraneoplastic syndrome in glomeruli, but are short of molecular details. When Lewis lung carcinoma 1 (LLC1) cells were implanted in mice lungs to establish lung cancer, renal failure was frequently observed two weeks post orthotopic xenograft. The high urinary albumin-to-creatinine ratio (ACR) was diagnosed as paraneoplastic nephrotic syndrome in those lung cancer mice. Profiling the secretome of the lung cancer cells revealed that the secretory proteins were potentially nephrotoxic. The nephrotoxicity of lung cancer-derived secretory proteins was tested by examining the pathogenic effects of 1 × 10^6^, 2 × 10^6^, and 5 × 10^6^ LLC1 cell xenografts on the pathogenic progression in kidneys. Severe albuminuria was present in the mice that received 5 × 10^6^ LLC1 cells implantation, whereas 10^6^ cell and 2 × 10^6^ cell-implanted mice have slightly increased albuminuria. Pathological examinations revealed that the glomeruli had capillary loop collapse, tumor antigen deposition in glomeruli, and renal intratubular casts. Since IL-6 and MCP-1 are pathologic markers of glomerulopathy, their distributions were examined in the kidneys of the lung cancer mice. Moderate to severe inflammation in the kidneys was correlated with increases in the number of cells implanted in the mice, which was reflected by renal IL-6 and MCP-1 levels, and urine ACR. TGF-β signaling-engaged renal fibrosis was validated in the lung cancer mice. These results indicated that lung cancer cells could provoke inflammation and activate renal fibrosis.

## 1. Introduction

Cancer is a malignant disease in which cells grow out of control, invade surrounding tissues, and spread to distant organs [1,2,3]. Uncontrolled cellular proliferation and metastasis are two deleterious phenotypes linked to cancer mortality [2,3]. In addition, malignant tumors can remotely injure other organs, which can also increase mortality [4]. Malignant tumors can also cause ectopic organ injury. Tumor lysis and paraneoplastic syndromes are two distinct ways by which malignant tumors can cause remote pathological progression in the kidneys [5,6]. Whereas tumor lysis syndrome is attributed to massive tumor cell death [6,7,8], paraneoplastic syndromes are caused by molecules secreted from tumor cells [9].

Chemotherapy, radiation therapy, or unknown massive cell death can also result in tumor lysis syndrome [7,10]. Clinically, tumor lysis syndrome is characterized by higher serum blood urea nitrogen (BUN), uric acid (UA), potassium, and phosphate, and low calcium levels in blood. The elevations in potassium, phosphate, and UA in the blood are caused by the dead cells releasing intracellular contents into circulating blood [7,8]. In contrast, paraneoplastic syndromes are pathogenic processes in which a malignant tumor indirectly induces pathophysiological events in organs far from the tumor itself [10,11,12,13].

Clinically, paraneoplastic syndromes are rare in cancer patients [5,13], and they may be silent or mild pathogenic processes without apparent syndromes in ectopic organs. Currently, the mechanism by which cancer cells remotely provoke pathological progression in distal organs is poorly understood. Through proteome profiling of secretomes, cancer cells have been shown to produce and secrete many proteins in culture [14]. These secretory molecules include extracellular proteins such as cytokines, growth factors, secretory enzymes and extracellular matrix-associated proteins, nuclear proteins, cytoplasmic proteins, and plasma membrane proteins [4,14]. These cancer cell-derived secretory proteins have been shown to be potential pathogenic inducers, triggering pathologic development in organs ectopically [15,16]. Clinically, small cell lung cancer produces vasopressin and induces syndrome inappropriate antidiuretic hormone (SIADH), and squamous cell carcinoma produces a variety of hormones and leads to paraneoplastic endocrine syndrome [4,9,17,18,19]. In addition, lung cancer cells have been shown to produce neuronal proteins to boost immune cells, and as a consequence, active immune cells attack the nervous system [20]. Moreover, lung cancer can also lead to pathological progression in the kidneys [5]. Clinical case reports have also documented that immune complexes or tumor antigens deposited in renal tubules or glomeruli can lead to glomerulopathy [18,21,22,23,24]. It is known that cancer cells are capable of the ectopic production and secretion of hormones, hormone-like molecules, cytokines, and immunoglobulins [13,15,16,25]. These molecules secreted by cancer cells are potentially toxic and can induce physiological dysfunction and organ failure.

Of the potentially nephrotoxic cytokines secreted by cancer cells, an animal model showed that the overproduction of Tumor Necrosis Factor (TNF) could lead to pathological progression in nude mice [15,16]. Consequently, hypercalcemia, leukocytosis, and muscle wasting as paraneoplastic disorders represent the pathogenic outcomes of TNF overproduction [16]. Pathologically, TNF overproduction may be linked to autoimmune responses and inflammation [26,27], which may not arise in athymic nude mice. Autoimmune-mediated nephropathy can occur in lung cancer patients when immune complex deposition, which consists of proteins secreted by lung cancer cells, accumulated in glomeruli, as this may locally boost immune cell activation [28,29]. Subendothelial, paramesangial deposits or deposits anywhere in the glomerular basement membrane (GBM) can lead to pathological progression and glomerulonephritis [20]. The pathomechanism by which lung cancer and other types of cancer induce paraneoplastic glomerulopathies or nephrotic syndromes is still unclear. Several studies and clinical case reports have described a similar phenomenon with the deposition of sediment in glomeruli that induces pathological events [4,22,30]. Apart from immune complex deposition, circulating inflammatory factors, which have also been identified in the cancer cell secretome, have also been reported to activate pathophysiological development in the kidneys, ultimately leading to End-Stage Renal Disease (ESRD) in patients with diabetes [31].

When we established lung cancer diseased C57BL/6 mice to evaluate effects of chemical medicaments on cancer growth and cancer-associated immune responses, we frequently observed unhealthy kidneys in the lung cancer mice. Particularly, cancer lysis and paraneoplastic nephrotic syndrome are two cancer-elicited kidney diseases. The two nephropathies can be discriminated by their serum and urine biochemical parameters. We observed that lung cancer mice are prone to kidney failure. Advancing in biochemical examination on renal function in the lung cancer mice, urine ACR is obviously elevated, and serum creatinine and potassium are slightly increased. Two weeks post orthotopic xenograft, the mice suffered from lung cancer. Additionally, kidney insult was observed. When we examined the kidneys of the lung cancer mice, the pathological features, including severe collapse of the glomerular capillary tufts and fibrosis, were impressively observed in the kidneys in the lung cancer mice. Profiling lung cancer secretome revealed that the lung cancer cell-derived secretory proteins are potentially nephrotoxic. To assess nephrotoxicity of LLC1 cells secreted factors, the conditioned medium from LLC1 cell culture was added to NRK-52E cells. Exposure of renal cells to LLC1 cell-conditioned medium resulted in IL-6 and MCP-1 expression in the NRK-52E cells. These results suggested that lung cancer-derived secretory proteins contribute to renal inflammation and fibrosis in the lung cancer mice as well as occurrence of paraneoplastic glomerulonephritis in lung cancer patients.

## 2. Results

### 2.1. The LLC1 Cells Grown in Mice Lung Caused Albuminuria

**Figure 1:**
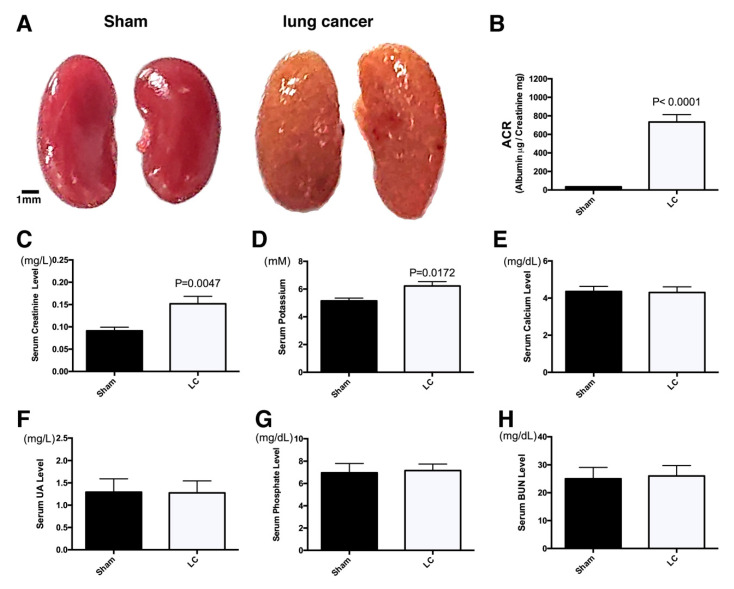
Orthotopic xenograft LLC1 (Lewis lung carcinoma 1) cells led to kidney injury. (**A**) Kidneys in the mice bearing lung cancer were pale and yellow-brownish in color anatomically (bar = 1 mm). (**B**) High urine ACR (albumin-creatinine ratio) was detected in the lung cancer mice. (**C**) Serum creatinine levels were slightly higher in the lung cancer mice than in the sham group. (**D**) After LLC1 implantation, the serum potassium level was slightly elevated. (**E**) Serum calcium levels were similar in the lung cancer and sham mice. (**F**) Serum uric acid (UA) levels were not elevated in the lung cancer mice. (**G**) The lung cancer and sham mice had similar serum phosphate levels. (**H**) The blood urea nitrogen (BUN) level was not obviously increased in the lung cancer mice.

LLC1 lung cancer cells were orthotopically implanted in C57BL/6 mice lung to establish the lung cancer animal model. Two weeks post orthotopic xenograft, a tumor grew in the left upper lobe of lung (Figure S1). The approximately two-thirds cross-sectional upper left lobe of the lung is lung cancer (Figure S2), so those lung cancer mice were assessed for higher tumor burden (Table S1). Besides the growth of lung cancer in the left upper lobe of mice lungs, we also observed unhealthy kidneys anatomically after the lung cancer mice had been sacrificed (Figure 1A). The kidneys in the lung cancer mice were pale and brownish-yellow compared to brown-reddish in the mice without tumor cell implantation (sham group), suggesting that kidney damage may have occurred. We then examined physiological parameters in sera and urine, including creatinine clearance, electrolyte homeostasis, and proteinuria, to determine the pathological causes.

To investigate and determine this pathology caused by either tumor lysis or the paraneoplastic effects, serum and urine that were collected from the lung cancer mice were subjected to biochemical analysis. Of physiological parameters, the albumin to creatinine ratio (ACR) in urine was much higher (733.7 ± 80 μg/mg) in the lung cancer mice than that (24.89 ± 1.8 μg/mg) in the sham group (Figure 1B). Additionally, serum creatinine and potassium levels were slightly elevated (0.15 ± 0.017 mg/L, and 6.22 ± 0.32 mM, respectively) in the lung cancer mice, compared to (0.09 ± 0.008 mg/L, and 5.15 ± 0.2 mM, respectively) the sham mice (Figure 1C,D). Otherwise, calcium, UA, phosphate, and BUN serum levels in the lung cancer mice were similar to the sham group (Figure 1E–H). These biochemical and physiological parameters are consistent with clinical cases documented in paraneoplastic nephropathy [20,21,32]. Anatomic observations of the kidneys revealed the obvious appearance of kidney injury two weeks after the LLC1 cells had been implanted into the mice lungs. A lower serum level of calcium and higher serum levels of phosphate, UA, and BUN were not found in blood samples from the lung cancer mice, indicating that the kidney failure was not attributed to massive tumor lysis (Figure 1E–H). The extremely high urine ACR and slightly elevated serum creatine and potassium levels in the lung cancer mice implied the occurrence of severe glomerular impairment and tubular lesions in their kidneys (Figure 1B–D).

### 2.2. The Collapse of Glomerular Tufts and Structural Lesions in Tubules Were Present in the Kidneys of the Lung Cancer Mice

**Figure 2:**
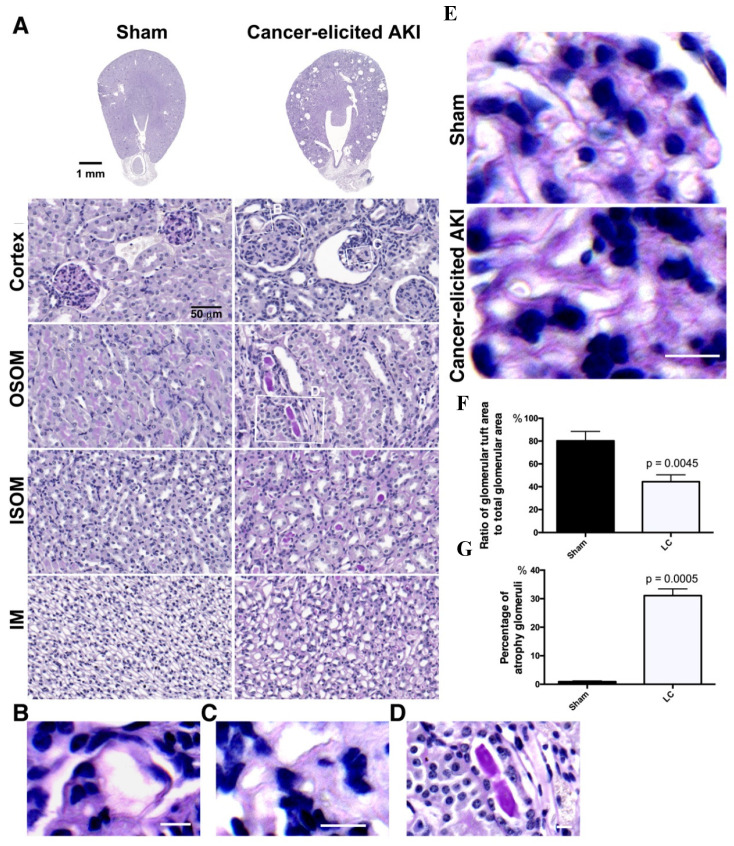
Histochemical examination of the kidneys of lung cancer mice. (**A**) Periodic acid Schiff (PAS) staining colored glycoproteins in brush borders in renal tubules and glomerular arteries. Histochemical imaging displayed impressive tuft collapse in glomeruli and atrophic glomeruli, brush border lesions in the outer stripe of the outer medulla (OSOM), casts in renal tubules in the OSOM, inner stripe of the outer medulla (ISOM), and inner medulla (IM) in the kidneys with acute kidney injury (AKI) in the lung cancer (LC) mice (bar = 50 μm). (**B**) Microscopic imaging with high power showed capillary dilation and hyaline-like material in arteriole lumen (bar = 10 μm). (**C**) PAS staining showed a monolayer capillary in a healthy kidney, whereas the capillaries in glomerulus were double contours in the lung cancer mice (bar = 10 μm). (**D**) The inset shows the presence of subendothelial deposits in the kidneys of lung cancer mice (bar = 10 μm). PAS-positive casting in renal tubules in the OSOM in the kidneys of lung cancer mice. (**E**) Glomerular capillaries with globular basement membrane (GBM) double contours were present in the glomerular tufts in the lung cancer mice. In contrast, a single layer of endothelial cells was seen in the glomerulus of healthy kidneys (bar = 10 μm). (**F**) The area of individual glomerulus and glomerular tufts was measured, and the ratio of glomerular tufts to glomerulus was calculated, as shown in healthy and lung cancer kidneys in the bar chart. (**G**) The numbers of healthy and unhealthy glomeruli in the kidneys were counted. The numbers of unhealthy glomeruli to total glomeruli were calculated in the sham and lung cancer mice, as shown in the bar chart.

As high urine ACR levels were detected in the lung cancer mice, impairment of glomerular structures and tubular function was suspected. Periodic acid Schiff (PAS) staining was performed to investigate the pathological characteristics of the kidneys in the lung cancer mice and examine glomerular capillary tufts and brush borders in renal tubules. Two weeks after tumor growth in the lungs of the LLC1 cell-implanted mice, collapse and wrinkling of glomerular tufts were observed in their kidneys (Figure 2A). Apart from glomerular damage, renal tubular lesions arising from brush border loss in the outer stripe of the outer medulla (OSOM) and PAS-positive casts in renal tubules in the OSOM and inner stripe of the outer medulla (ISOM) were seen (Figure 2A, Figures S3 and S4).

On further examination of the renal damage, ordered organization of capillary tufts in glomeruli were seen and magenta-stained brush borders remained in the proximal tubules in the OSOM of healthy kidneys. In contrast, collapsed tufts with occlusion of the capillary lumina, enlarged capillary loops, dilated capillaries, double contours of many basement membranes, and the presence of dilated capillaries filled with a hyaline-like material and subendothelial deposits were clearly seen in glomeruli (Figure 2B–E).

The glomerular tufts with collapsed glomerular basement membrane (GBM) were described as the ratio of glomerular tuft area to glomerular volume, which was calculated to be 80.23% in the healthy mice and 44.46% in the lung cancer mice (Figure 2F). We then quantified glomerulopathy according to the percentage of collapsed glomerular tufts in the total number of glomeruli, and found a mean 31.08% ± 0.236 atrophic glomeruli in kidneys of lung cancer mice, compared to 0.916% ± 0.127 in the healthy mice (Figure 2G).

### 2.3. Proteins Identified in Lung Cancer Cell Secretome Potentiated Nephrotoxicity

**Figure 3:**
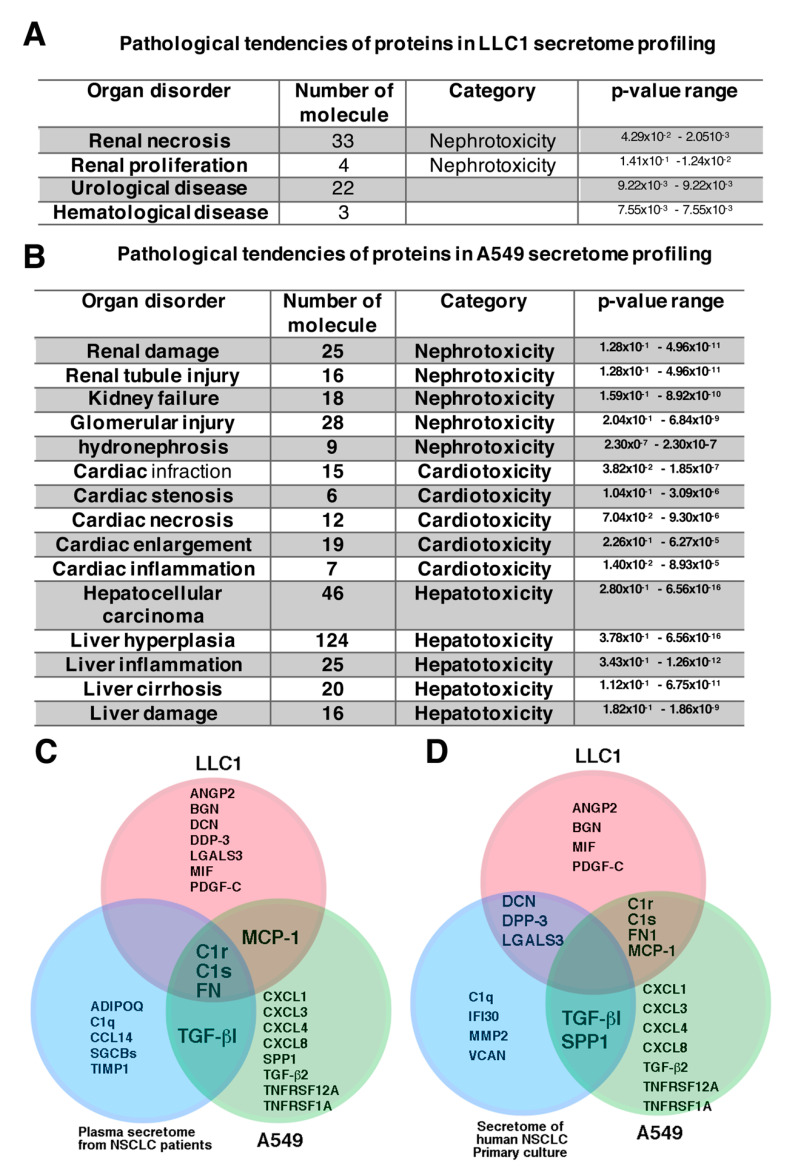
Ingenuity pathway analysis of the proteins identified in lung cancer cell secretomes. (**A**) Classification of the involvement of LLC1 cell-derived secreted proteins in various diseases. (**B**) Classification of the involvement of A549 cell-derived secreted proteins in various diseases. (**C**) Overlap of proteins identified in the LLC1 and A549 cell secretomes in the present study and serum secretome of non-small cell lung cancer (NSCLC) patients by Shin et al. [34]. (**D**) The commonly and differentially expressed secretory proteins in LLC1 cells and A549 cells in the present study and in the human NSCLC primary culture reported by Feng et al. [33].

In general, studies on tumor cell secretomes have mainly focused on identifying tumor markers to aid in making a diagnosis and estimating prognosis [14,33,34]. However, the proteome profiling of a secretome can also provide pathological information related to paraneoplastic disorders in kidneys. Proteome profiling of the LLC1 cell secretome identified 594 proteins in the conditioned medium in which the LLC1 cells grew (Table S3). Additionally, 202 proteins were identified in the secretome from the A549 cell-conditioned medium (Table S4). Ingenuity pathway analysis (IPA) of the proteome of the lung cancer secretome predicted that the proteins secreted by LLC1 cells potentiated nephrotoxicity (Figure 3A), whereas pathway analysis of the A549 lung cancer secretome predicted that the proteins secreted by A549 lung cancer cells potentiated nephrotoxicity, cardiotoxicity, and hepatotoxicity (Figure 3B). Although it has been documented that the deposition of antigens expressed by tumor cells in glomeruli may be a pathogenic cause of renal injury and kidney failure [22,24], the immunoglobulins reported to be deposited in the glomerular mesangial area, such as IgA and IgG [35,36], were not present neither in LLC1 nor A549 secretome profiling. Among the extracellular proteins, C1s, C1r, and fibronectin, which are involved in renal fibrosis, were proteomically identified in both the LLC1 and A549 cell secretomes in the present study (Figure 3C, Tables S3 and S4), which is consistent with the study by Shin et al. [34]. None of the common nephrotoxic secretory proteins were identified in either the LLC1 or A549 secretome in the present study or in a human non-small cell lung cancer (NSCLC) primary culture secretome reported by Feng et al. [33] (Figure 3D). Among the LLC1-derived nephrotoxic secretory proteins, ANGPL2, BMP-1, DCN, FN1, and MCP-1 have been shown to contribute to TGF-β signaling-dependent renal fibrosis (Table 1) [37,38,39,40,41]. These nephrotoxic secretory proteins are not only immune-related proteins and cytokines, but also growth factors, enzymes, and extracellular matrix-associated proteins (Table 1 and Table S2).

### 2.4. Fewer LLC1 Cells Implanted in Mice Develop Relatively Mitigaged Renal Insult and Inflammation

The IPA to predict lung cancer secretome-associated nephrotoxicity indicated renal fibrosis, inflammation, and glomerulopathy might occur as lung cancer secretory proteins are constantly stimulating to renal cells. IL-6 and MCP-1 have been reported to be pathogenic markers of renal inflammation with albuminuria in patients with a variety of kidney injuries [41,54,55], and thus expressions of IL-6 and MCP-1 were selectively investigated in the kidneys of those lung cancer mice. The degree of pathological severity and albuminuria in the lung cancer mice may correspond to the amount of protein secreted by the LLC1 cells. A decreasing number of LLC1 cells implanted in the lung may induce mild pathological effects of secretome-associated nephrotoxicity, if the proteins secreted by cancer cells cause renal insult. Therefore, we orthotopically implanted 1 × 10^6^, 2 × 10^6^, and 5 × 10^6^ cells into the left upper lobe lung of individual mice.

**Figure 4:**
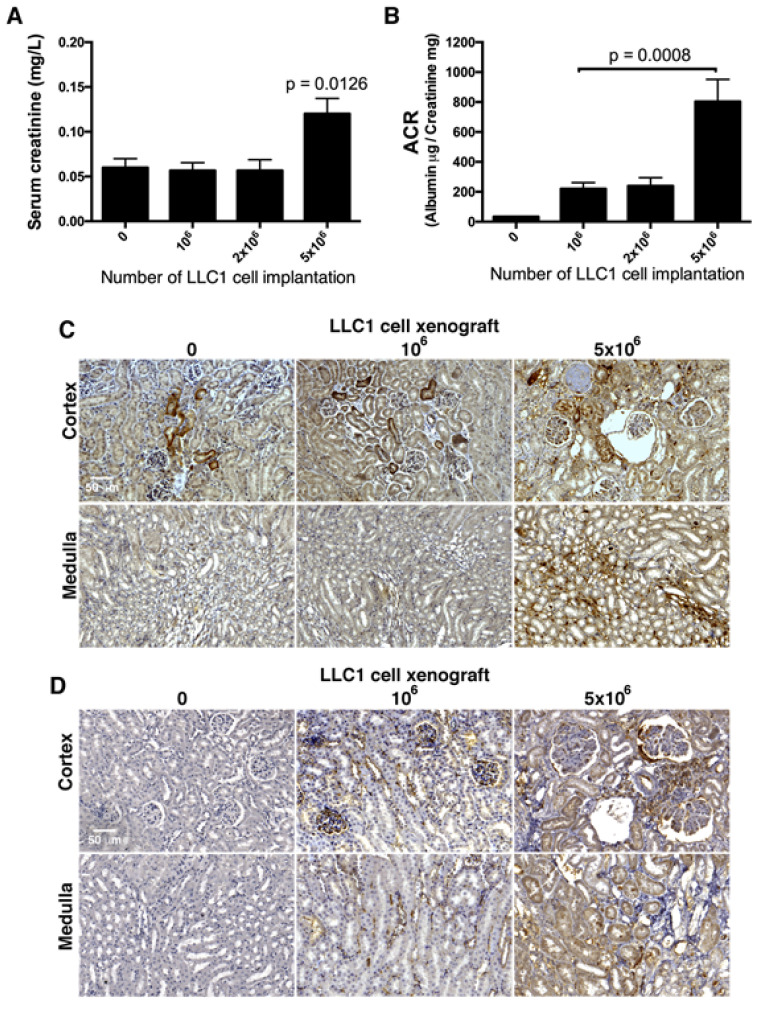
The number of LLC1 cells transplanted in the lungs was correlated with the severity of paraneoplastic inflammation in the kidneys. (**A**) Serum creatinine levels were not obviously increased in the mice with 1 × 10^6^ and 2 × 10^6^ LLC1 cells, and were slightly elevated in the mice with 5 × 10^6^ LLC1 cells. (**B**) The urinary albumin to creatinine ratio (ACR) was slightly increased in the mice with 1 × 10^6^ and 2 × 10^6^ LLC1 cells, and robustly elevated in the mice with 5 × 10^6^ LLC1 cells. (**C**) Renal inflammation was detected using immunohistochemical staining with specific antibody-recognized MCP-1. Relatively fewer renal tubules in the cortex and medulla had MCP-1 staining in the sham group, whereas more MCP-1 staining was seen in the renal tubules in the cortex, but not medulla in the mice with 1 × 10^6^ LLC1 cells, and a large increase in MCP-1 staining was detected in the cortex and medulla in the mice with 5 × 10^6^ LLC1 cells. (**D**) IL-6 as a proinflammatory cytokine was detected in the kidneys in both the lung cancer and sham mice. A few IL-6 signals were displayed in the kidneys of the sham mice, whereas IL-6 expression was relatively higher in the kidneys of the mice with 1 × 10^6^ LLC1 cells, and IL-6 signals were strongly displayed in the kidneys of the mice with 5 × 10^6^ LLC1 cells. Bar = 50 μm.

Two weeks after LLC1 cell implantation in the mice lungs, serum creatinine and urine ACR were measured as renal function indexes. The mean serum creatinine levels were 0.6 ± 0.0057 mg/dL, 0.57667 ± 0.088 mg/dL, and 0.5667 ± 0.12 mg/dL, and not obviously elevated in the sham, 1 × 10^6^, and 2 × 10^6^ cell xenograft groups, respectively. In comparison, the serum creatinine level was higher (mean 1.2 ± 0.1732 mg/dL) in the 5 × 10^6^ cell xenograft mice than in the mice with fewer lung cancer cells (Figure 4A). The mean urine ACRs, which is a marker of glomerular lesions, were 302.4 ± 41.36 μg/mg and 301 ± 55.19 μg/mg in the 1 × 10^6^ and 2 × 10^6^ LLC1 cell groups, 803.7 ± 147.4 μg/mg in the 5 × 10^6^ group, and 33.87 ± 1.24 μg/mg in the sham group (Figure 4B).

Investigating renal inflammation in the lung cancer mice, the distributions of IL-6 and MCP-1 in the kidneys were evaluated by immunohistochemical staining. As noted in immunohistochemical imaging, IL-6 and MCP-1 were both extremely elevated in kidneys of the mice implanted with 5 × 10^6^ LLC1 cells. Observation on IL-6 expressions displayed that the IL-6 was sporadically distributed in a few renal tubules in cortex and medulla in kidney in the sham mice, and relatively higher in the renal tubules in cortex in 1 × 10^6^ LLC1 cell-implanted lung cancer mice (Figure 4C). Then, MCP-1 was almost nondetectable in kidneys of the sham mice, and relatively higher in renal tubules in cortex and tubulointerstitial space of renal tubules in the medulla in the kidneys of the mice with 1 × 10^6^ LLC1 cell implantation (Figure 4D). More severe inflammation was noted in the kidneys of the mice with 5 × 10^6^ LLC1 cells. In particular, both IL-6 and MCP-1 were present in the margins of glomerular tufts in damaged glomeruli and in pericytes surrounding the renal tubules (Figure 4C,D).

### 2.5. Mice Transplanted with Fewer LLC1 Cells Had Milder Renal Fibrosis

**Figure 5:**
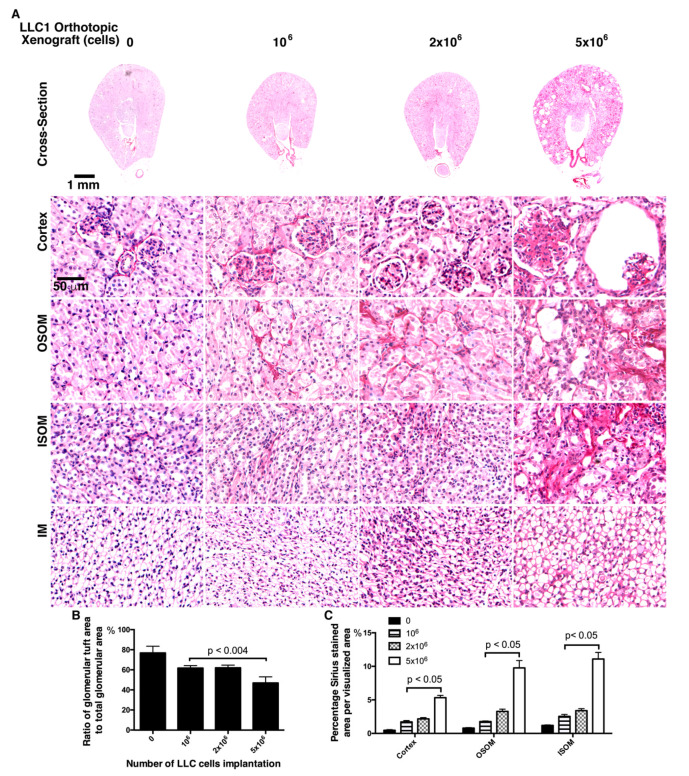
Renal fibrosis in the kidneys of lung cancer mice. (**A**) Renal fibrosis was detected in mice transplanted with different numbers of LLC1 cells by Sirius red staining. The Sirius red-labeled collagen protein was used to assess the grade of renal fibrosis. Few Sirius red signals were detected in the kidney without LLC1 xenografts. There were slightly more Sirius red-stained collagen proteins in the cortex, outer stripe of the outer medulla (OSOM), and inner stripe of the outer medulla (ISOM) in the kidneys of mice with 1 × 10^6^ and 2 × 10^6^ LLC1 cells. Severe renal fibrosis was noted in the cortex, OSOM, and ISOM in the kidneys of mice with 5 × 10^6^ LLC1 cells (bar = 50 μm). (**B**) Glomerular impairment was represented as a lower ratio of glomerular tufts to total glomerulus (bar chart of the ratio of glomerular tufts to total glomerulus). (**C**) The Sirius red-stained area was measured, which showed severe renal fibrosis.

The IPA prediction of proteomics of lung cancer secretome has predicted that renal fibrosis as pathogenic relevance of nephrotoxic molecules in lung cancer cell secretome. Additionally, IL-6 and MCP-1 expressions in kidney is coincident with renal fibrosis [56,57]. To assess renal fibrosis in the lung cancer mice, the Sirius red staining allowed visualization of collagen distribution and validation of renal fibrosis. As noted in Sirius red staining, few collagenous proteins accumulated in the cortex, OSOM, or ISOM in the kidneys of the mice without tumor cell implantation (Figure 5A). In the 1 × 10^6^ and 2 × 10^6^ LLC1 cell-implanted mice, more Sirius red staining was present in the cortex, OSOM, and ISOM, and severe renal fibrosis was noted in glomeruli in the cortex and tubulointerstitial space in the OSOM and ISOM of mice with 5 × 10^6^ LLC1 cells (Figure 5A). In addition, glomerular nephropathy was evaluated in the glomerular tufts in the glomeruli (Figure 5B,C). The ratio of glomerular tufts to glomerular volume was calculated, and statistical analysis showed approximately 80% glomerular tufts in all glomeruli in the sham mice, approximately 60% in the mice with 1 × 10^6^ and 2 × 10^6^ LLC1 cells, and 45% in the mice with 5 × 10^6^ LLC1 cells (Figure 5C). Additionally, the degree of renal fibrosis was examined using Sirius red staining in the mice with different numbers of LLC1 cells. The results showed that more tumor cell growth in the lungs exacerbated the progression of renal fibrosis in the kidneys and caused a greater number of lesions in the glomeruli (Figure 5C).

### 2.6. Activation of TGF-β Signaling in the Kidneys of the Lung Cancer Mice

**Figure 6:**
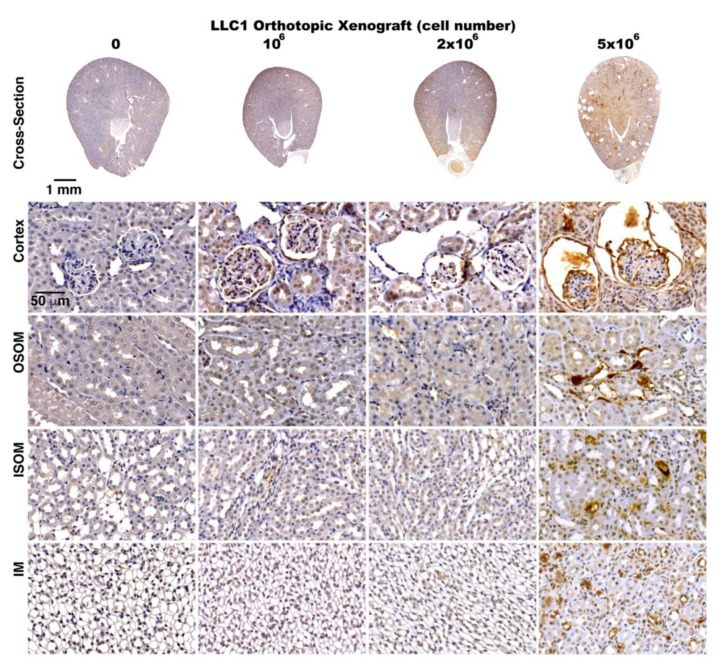
TGF-β as a pathogenic signaling of renal fibrosis in the kidneys of lung cancer mice. The role of TGF-β signaling in paraneoplastic renal fibrosis in the kidneys of the mice with LLC1 cells was evaluated using an antibody against TGF-β to determine TGF-β distribution in the kidneys. Fewer TGF-β signals were detected in the sham mice compared to increasing signals in the mice with increasing numbers of LLC1 cells. More TGF-β signals were noted in the cortex, outer stripe of the outer medulla (OSOM), inner stripe of the inner medulla (ISOM), and inner medulla (IM) in the kidneys of the lung cancer mice. Bar = 50 μm.

TGF-β signaling has been shown to be a pathological pathway involved in renal fibrosis in kidneys exposed to renal insults, such chemotherapeutic agents or nephrotoxins [57]. Concerning renal fibrosis, proteins secreted by lung cancer cells could lead to TGF-β signaling in kidneys (Table 1 and Table S2). Along with renal fibrosis in the lung cancer mice, the role of TGF-β signaling in renal fibrosis was verified by immunohistochemical staining with an antibody specifically recognizing TGF-β. Robust TGF-β signals were detected in the cortex, OSOM, and ISOM in the kidneys of the mice with 5 × 10^6^ LLC1 cells, and mild TGF-β signals were noted in the kidneys of the mice with either 1 × 10^6^ or 2 × 10^6^ LLC1 cells (Figure 6). Very intense TGF-β signals were detected in glomeruli of the lung cancer mice (Figure 6). These results indicated that the cells in glomeruli were susceptible to lung cancer cell-derived toxic stimulation.

### 2.7. Cancer Secretome-Activated Renal Inflammation

**Figure 7:**
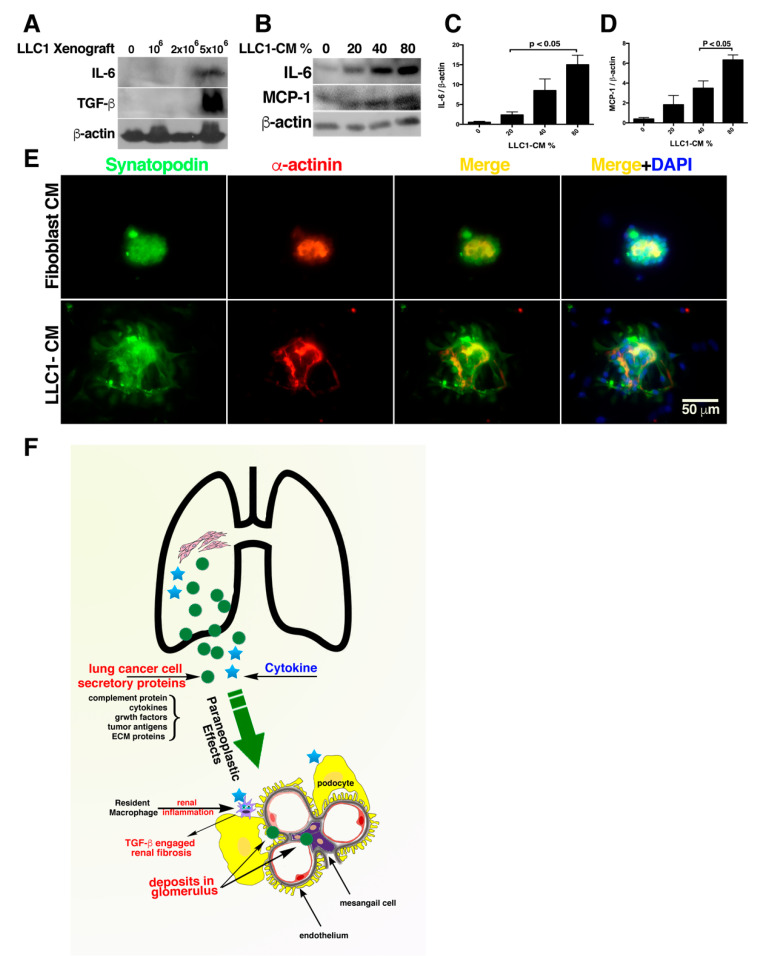
LLC1 cell-conditioned medium (LLC1-CM) provoked inflammation in renal cells and elicited glomerular integrity. (**A**) Western blot analysis showed elevated IL-6 and TGF-β expressions in the kidneys of the lung cancer mice. The whole gel figures are shown in Figure S5. (**B**) The LLC1-CM provoked inflammation in the kidneys. As 20%, 40%, and 80% conditioned medium was added to NRK-52E cell cultures, the IL-6 and MCP-1 expressions increased. (**C**) IL-6 expression level in NRK-52E cells was calculated as the ratio of IL-6 to β-actin. IL-6 levels were relatively higher when the cells were exposed to conditioned medium collected from LLC1 cell culture. (**D**) When NRK-52E cells were exposed to LLC1 cell-derived conditioned medium, the MCP-1 expression was obviously increased. (**E**) Exposure of glomeruli to LLC1-conditioned medium (LLC1-CM) leads to loss of glomerular integrity and the podocytes in the glomerulus do not arrange in cluster-form. In contrast, when glomerulus cultured in fibroblast-conditioned medium (fibroblast CM), podocytes tightly cluster (bar = 50 μm). (**F**) Schematic representation depicted a plausible pathogenic mechanism associated with glomerulopathy in the lung cancer mice.

To evaluate inflammation and the role of TGF-β in renal fibrosis in the kidneys of lung cancer mice, the kidney tissues were subjected to Western blot analysis. The kidneys of the lung cancer mice expressed relatively more IL-6, particularly those of the mice transplanted with 5 × 10^6^ LLC1 cells (Figure 7A–C). In addition, the TGF-β levels were robustly elevated in the kidneys of the mice transplanted with 5 × 10^6^ LLC1 cells (Figure 7A).

To test the pathogenic effects of lung cancer secretome on renal inflammation, conditioned media that were collected from LLC1 cell cultures were added to cultured renal cells NRK-52E. After stimulation with the conditioned media, the NRK-52E cells expressed much higher levels of MCP-1 and IL-6 compared to very low IL-6 and MCP-1 expressions in the NRK-52E cells without conditioned medium stimulation (Figure 7B–D).

### 2.8. Exposure of Glomeruli to LLC1 Cell-Conditioned Medium Leads to Loss of Glomerular Integrity

As cells in glomeruli are susceptible to lung cancer cells secretome-elicited toxic stimulation, the pathological aspect in glomeruli might be observed after exposure of glomeruli to LLC1 cell-conditioned medium. The glomeruli that were isolated from C57BL/6 kidneys cultured in Roswell Park Memorial Institute (RPMI) 1640 medium, those podocytes remain in cluster-form within 96-h culture, as those podocytes stand in a glomerular integrity. To test nephrotoxicity of LLC1 cell-conditioned medium (LLC1-CM), the glomeruli that were cultured in RPMI medium for 48 h were exposed to 20% LLC1-CM for 18 h. After exposure of glomeruli to LLC1-CM, podocytes were stained with antibody-recognized synaptopodin and antibody-recognized α-actinin, since interaction of synaptopodin and α-actinin regulates microfilament bundling in the foot processes of podocytes [58]. As noted in the glomeruli exposed to mouse embryo fibroblast (MEF)-derived and conditioned medium (Fibroblast CM), the podocytes form a cluster-like pattern (Figure 7E). When glomeruli were incubated in 20% LLC1-CM for 18 h, not all podocytes arranged in cluster-form. Some of them, however, still retained ability for cell adhesion, and were no longer tightly adhering to one another as the podocytes in the glomerulus developed a loss of glomerular integrity (Figure 7E).

## 3. Discussion

Paraneoplastic syndrome is a cancer-related complication. There are currently few systematic investigations and a lack of experimental evidence of its pathogenic causes and mechanisms at a molecular level. Clinically, biopsies can only offer limited knowledge on paraneoplastic nephrotic syndrome. A diseased animal model may be able to elucidate the pathological mechanism at a molecular level; however, such models are currently not available. The lung cancer-diseased animal model, the C57BL/6 mice with LLC1 cell orthotopic xenograft, had pale kidneys, indicating a renal insult from a paraneoplastic disorder (Figure 1A,B). Zhang et al. subcutaneously implanted LCC1 cells in C57BL/6 mice, and found that this could activate Toll-like receptor 4 (TLR-4) to drive proteasome and changes in muscle catabolism [59]. Muscle wasting is counted to a paraneoplastic syndrome, implying that the LLC1 cells in the C57BL/6 mice could potentially have provoked paraneoplastic syndrome. Muscle wasting may also cause renal failure, as rhabdomyolysis-induced kidney injury has been reported in an animal model and clinical cases [60]. Azotemia with elevation of BUN is a pathological indicator of rhabdomyolysis-induced renal failure [61]. In the present study, serum BUN was not obviously elevated in the lung cancer mice (Figure 1H). Therefore, this rhabdomyolysis may not be a pathological cause of renal failure in LLC1 cell orthotopic xenografted mice. It seems that kidney failure in the lung cancer mice was caused by another paraneoplastic stimulation.

Two weeks after the implantation of 5 × 10^6^ LLC1 cells, lung cancer grew orthotopically and severe kidney disease developed in the mice (Figure 1A,B and Figure S2). As the cancer cells secreted proteins that were involved in the pathological progression of paraneoplastic syndromes in the kidneys, the implantation of fewer cells may have elicited mild or moderate syndromes. When fewer LLC1 cells (1 × 10^6^ and 2 × 10^6^) were implanted in the lungs, renal damage was relatively less severe. In addition, urine ACR, renal inflammation, and fibrosis were moderate in the 1 × 10^6^ and 2 × 10^6^ LLC1 cell groups (Figure 4A–D, Figure 5A–C, and Figure 6). This suggests that secretions from fewer than 2 × 10^6^ LLC1 cells did not reach a pathogenic dose to induce severe paraneoplastic nephrotic syndrome.

How cancer cells remotely induce renal injury is not yet clearly understood. Two clinical studies reported that cancer-derived secretory proteins potentially have a pathogenic impact on organ damage ectopically [17,21]. Although tumor cell-derived secretory proteins are regarded as a pathogenic inducer contributing to paraneoplastic nephrotic syndrome (PNS) are not yet characterized exactly what molecules act the pathogenic role in PNS. To estimate the pathological effect of proteins secreted by lung cancer cells, secretomes of the conditioned medium in which LLC1 cells and A549 cells grew were profiled by mass spectrometry-based secretome profiling (Figure 3A and Table S3). IPA of the proteome in the LLC1 cells and A549 secretomes predicted their pathological potential for a variety of renal injuries, including renal necrosis and proliferation (Figure 3A,B). When the renal cells exposed to LLC1 cell-conditioned medium as well respond upon LLC1 cell-derived pathogenic factors in vitro, the renal cells produced IL-6 and MCP-1, as they suffered from inflammation (Figure 7B–D). In addition, the primary culture glomeruli in the LLC1-CM results in loss of glomerular integrity (Figure 7D). This suggests that lung cancer cells could drive nephropathy via pathogenic stimulation by their secretory proteins. The nephrotoxic factors in LLC1 cell secretome consist of cytokine, complement proteins, growth factors, and extracellular matrix (ECM)-associated proteins.

Of proteins in LLC1 secretome profiling (Figure 3A,C,D and Table 1), the PNS could be attributed to growth factor-induced pathogenic development, complement proteins trapped in the kidney that trigger a disordered immune reaction, cytokine-stimulated inflammation, immune cell-attacked tumor antigens that are secreted by tumor cells and accumulated in glomeruli or renal tubules, or ECM-associated protein deposited in renal interstitial and glomeruli to direct nephropathy in the kidney (Figure 7F). Histologically, collapse of glomerular capillary tufts was a striking pathological pattern in the lung cancer mice (Figure 2A). Similar pathological features were reported in the pathological examinations of kidney biopsies in patients with glomerulonephritis [20,62]. The glomerulonephritis arises in glomeruli with immunoglobulin deposition, which immune cells attack [62,63,64,65]. Histochemical imaging has shown a GBM with double contours, collapse of glomerular tufts, and subendothelial deposits in glomerulonephritis [20,62]. Intriguingly, although immunoglobulins were not found in the lung cancer secretome (Tables S3 and S4), cytokines and complement C1r and C1s that were identified in the lung cancer secretome are potential triggers of nephropathy (Figure 3C and Table 1).

Shin et al. reported serological markers of non-small cell lung cancer (NSCLC) lung cancer patients, the C1r, C1s, and fibronectin are the proteins identified in LLC1 cells and A549 in the present study, and serum secretome of NSCLC lung cancer patients [34]. Interestingly, the three proteins were also identified in several cancer cell-derived secretomes [14,33,34]. Complement deposition in the kidney results in activating the lectin pathway and driving aberrant immune activation in the kidney, and lead to glomerulopathy [66,67,68]. The pathogenic role of fibronectin in renal insult, such as mesangial expansion, glomerulopathy, and proteinuria, was reported [40,47,69]. Additionally, cytokines, such as MCP-1 and MIF, were identified in LLC1 cell-derived secretome (Figure 3C and Table 1). Niewczas et al. reported that TNF-1AR is involved in the pathological progression to End-Stage Renal Disease (ESRD) in patients with diabetes [31]. These proinflammatory factors may act as pathogenic inducers to exacerbate pathophysiological development in kidneys. Although the molecules released by lung cancer cells that lead to renal failure in patients with lung cancer, as well as mice with LLC1 cell xenografts, have yet to be experimentally demonstrated, it has been documented in clinical studies and animal models that the same proteins in circulation are able to drive nephropathy without cancer growth.

Approximately 1% of lung cancer patients develop severe kidney disease. Cancer cell-elicited nephrotic syndrome might initiate the cancer cell secretory proteins as depositions that are trapped in glomeruli and exacerbate kidney injury by regular stimulation of proinflammatory factors, which the lung cancer cells release to circulation, and overreactive immune cells attacking tumor antigen deposition in the kidney [21,22]. As a tumor grows, tumor cells secrete proteins into the blood circulation, and the deposition of these cancer antigens in glomeruli could be the pathological cause of paraneoplastic nephropathy [36,70]. The cancer antigen deposition present in the kidney, as similar to immune complex deposition accumulated in glomeruli, could be a target for immune cells. Additionally, aberrant immune activation can also contribute to disordered immune reaction to induce kidney failure. The deposition of cancer antigens in glomeruli may also be exacerbated in cancer patients who undergo immune therapy. We speculate that immune cells may attack tumor cells and coincidently attack the glomeruli that have captured cancer antigens as protein deposits in patients receiving immune therapy. As a consequence, paraneoplastic nephropathy would be more pronounced in cancer patients who receive immune therapy. Our proposed animal model for paraneoplastic syndrome in kidneys may offer useful information for clinicians and cancer biologists.

## 4. Material and Methods

### 4.1. Materials

Detailed materials information is listed in the key resources, Table S5.

### 4.2. Animal Care and Lung Cancer Cell Orthotropic Xenograft Model

C57BL/6 mice were purchased for animal experiments in this study (Charles River Technology, Bio-LASCO Taiwan Co. Ltd., Taipei, Taiwan). The animals were maintained at room temperature (22 ± 2 °C), with 50 ± 10% humidity and an automatically controlled cycle of 12 h light and 12 h dark. All experimental procedures and housing were approved by the Institutional Animal Care and Use Committee (IACUC) of Kaohsiung Medicine University (KMU) (Document: 107244). To establish an orthotopic animal model of lung cancer, mouse Lewis lung carcinoma cells 1 (LLC1) were transplanted into the left upper lobe of the lungs in the C57BL/6 mice. Two weeks after LLC1 transplantation, the mice were sacrificed. Kidneys and lungs were collected and placed in aqueous formaldehyde solution (37% *w*/*w*) containing sodium phosphate to provide buffering to an approximately isotonic solution with pH 7.2–7.6 for 24 h.

### 4.3. Biopsy Collection, Histochemical Staining, and Immunohistochemical Staining

The kidney and lung tissues that were preserved in aqueous formaldehyde solution for 24 h were dehydrated and embedded in paraffin blocks. The paraffin blocks were sliced at 4-μm thickness with a sliding microtome (SM2125, Leica Biosystems, Nussloch, Germany). For histochemical examinations, the specimens were deparaffinized in xylene and sequentially rehydrated using 100% ethanol, 90% ethanol, 70% ethanol, and water. To investigate pathological features of the kidneys, periodic acid Schiff (PAS) (Sigma/Merck, Darmstadt, Germany) staining was carried out to colorize brush borders and glomerular capillaries. To examine fibrosis, the specimens were subjected to Pico Sirius-red (Merck, Darmstadt, Germany) staining. The kidney specimens were immunohistochemically stained with TGF-β1 (Arigo Laboratories, Hsinchu, Taiwan), MCP-1 (ABclonal, Woburn, MA, USA), and IL-6 (ABclonal, Woburn, MA, USA) antibodies. The distributions of TGF-β1, IL-6, and MCP-1 in the kidneys were detected using commercially available peroxidase IHC kits, and developed with diaminobenzidine (DAB) (ThermoFisher, Waltham, MA, USA). The histochemical and immunohistochemical specimens were microscopically scanned and digitalized with a Pannoramic MIDI digital scanner (3DHISTECH Ltd., Budapest, Hungary). Microscopic photos were acquired using Pannoramic Viewer 1.14 (3DHISTECH Ltd., Budapest, Hungary).

### 4.4. Evaluation of Serum Physiological Parameters

Serum levels of UA, creatinine, and BUN were measured using a Roche cobas 600c 501 analyzer (Roche Diagnostics, Manheim, Germany). Serum sodium level and osmolality were determined using a Roche cobas b121 POC system ion-selective electrode (Roche Diagnostics, Basel, Switzerland).

### 4.5. Cell Culture and Orthotopic Xenograft

LLC1 cells (obtained from American Type Culture Collection (ATCC, Manassas, VA, USA) were applied to establish an orthotopic lung cancer model in the C57BL/6 mice. The LLC1 cells were cultured in Dulbecco’s Modified medium (DMEM) (ThermoFisher, Waltham, MA, USA) supplemented with 10% fetal bovine serum (ThermoFisher, Waltham, MA, USA), 2 mM glutamine (ThermoFisher, Waltham, MA, USA), 100 U/mL penicillin, and 0.1 mg/mL streptomycin (ThermoFisher, Waltham, MA, USA) at 37 °C in a humidified atmosphere containing 5% CO_2_. For the xenograft experiments, 5 × 10^6^, 2 × 10^6^, and 10^6^ cell suspensions were prepared in 20 μL serum-free DMEM medium, and orthotopically injected into the right upper lobe of mice lungs, individually. Mice with LLC1 xenografts (LLC1 mice) were sacrificed on the 14th day post LLC1 transplantation.

### 4.6. Cell Culture and Proteomic Profiling of Secretome

A549 cells were purchased from ATCC. The cells were cultured in RPMI 1640 medium supplemented with 10% fetal bovine serum at 37 °C in a humidified atmosphere at 5% CO_2_. To profile the secretome, cells were cultured in serum-free RPMI for 72 h. The conditioned medium was collected, and proteins in 20 mL of the medium were precipitated with 50% ammonium sulfate (Merck, Darmstadt, Germany). The proteins were then centrifuged at 4000× *g* for 30 min at 4 °C, and the protein pellet was dissolved in 1 mL Tris-HCl buffer (20 mM Tris-Cl at pH 8.0). The protein solution was then transferred to dialysis tubing (ThermoFisher, Waltham, MA, USA) with a molecular weight cutoff (MWCO) of 8000–6000 Da, and dialysis was carried out to discard remnant ammonium sulfate in the protein solution. After dialysis, the protein solution was brought up to 2 mL, and secretome proteins (10 μg) were subjected to proteomic analysis.

### 4.7. Cell Culture and Secretome-Induced Inflammation

NRK-52E cells (purchased from ATCC) were cultured in DMEM medium supplemented with 10% fetal bovine serum at 37 °C in a humidified atmosphere at 5% CO_2_. To test the potential of the secretome to stimulate inflammation, 10^5^ NRK-52E cells were seeded in a 100 mm Petri dish and grown for 48 h. The conditioned medium in which the LLC1 cells had been incubated was collected, and 20%, 40%, and 80% conditioned medium samples were prepared. The cells were then incubated in 0%, 20%, 40%, and 80% conditioned medium, respectively. After incubation for 24 h, the cells were collected and cell lysates subjected to Western blot analysis.

### 4.8. Renal Cells Primary Culture

Kidneys were removed from C57BL/6 mice. Cortex and medulla were separated. Renal cortex was minced and soaked in serum-free RPMI medium. The minced renal cortex was gently sieved through a 70 μm strainer and the individual glomerulus was sieved out of the medium using the 70 μm strainer. The free glomeruli were collected with a 30 μm strainer, and then glomeruli in the strainer were flushed out with RPMI medium. Glomeruli in RPMI medium were counted and two hundred glomeruli were seeded on 5% gelatin-coated, 12 mm coverslip in a 24 well plate. Whole glomeruli were cultured in RPMI supplemented with 10% FBS at 37 °C in a humidified atmosphere at 5% CO_2_. For primary culture of renal tubular epithelial cells, the renal medulla was minced and digested in type IV collagenase (1 mg/mL in 1×Hanks’s Balanced Salt Solution (HBSS)) (Sigma-Aldrich, St. Louis, MO, USA). After digestion, renal tubular epithelial cells were centrifuged at 300 *g* for 5 min. The cells pellet was resuspended in RPMI medium, and cell number was counted with hemocytometer. The 10^4^ cells were seeded on a 12 mm coverslip in a 24 well plate. The cells were incubated at 37 °C in a humidified atmosphere at 5% CO_2_.

### 4.9. Glomerular Integrity Assay

After glomeruli are docked on the gelatin-coated coverslip, podocytes form a cluster within 96 h of glomeruli culture. To assess pathological effect of LLC1 cell-derived conditioned medium, the glomeruli seeded on the coverslip were incubated in the RPMI with 20% conditioned medium for 18 h. To test nephrotoxicity, exposure of glomeruli to 20% conditioned medium for 18 h were settled. Following this, the glomeruli were harvested and fixed in 4% paraformaldehyde in CSK buffer (10 mM Pipes, 100 mM NaCl, 3 mM MgCl_2_, 1 mM EGTA, 300 mM sucrose, pH 6.8) for 10 min, and permeabilized in 0.5% Triton in CSK buffer for 5 min. After fixation. the cells in the glomeruli were stained with the antibody-recognized synatopodin and antibody-against α-actinin. Glomerular integrity was validated as the podocytes form a cluster. In contrast, loss of glomerular integrity displays podocytes detachment from the podocytes-clustered glomerulus.

### 4.10. Immunofluorescent Staining and Immunofluorescent Microscopic Imagination

A Leica DMi8S epifluorescence microscope (Leica Microsystem, Wetzlars, Germany) equipped with X-Cite XCT10A (Lumen Dynamics, Wiesbaden, Germany) light source, filters, and 63× objective was used to observe the cells in glomeruli. The antibodies-recognized synatopodin (1:500, Santa Cruz Biotechnology, Santa Cruz, CA, USA) and α-actinin (1:500, Santa Cruz Biotechnology, Santa Cruz, CA, USA) were applied in immunofluorescent staining for podocytes in glomeruli. An additional reagent used was DAPI (0.2 μg/mL 4′,6-diamidino-2-phenylindole, Sigma-Aldrich), for staining cell nuclei.

### 4.11. Immunoblot

Kidney tissues were collected and homogenized in 1× RIPA buffer (ThermoFisher, Waltham, MA, USA) containing protease inhibitors and phosphatase inhibitors (Biotools, New Taipei City, Taiwan) with Dounce homogenizer. The protein concentration was determined using a Pierce BCA protein assay kit (ThermoFisher, Waltham, MA, USA). Total kidney proteins (30 μg) were migrated in SDS-PAGE and transferred to a PVDF membrane (Merck, Darmstadt, Germany). The membranes were blocked in 5% skim milk for 2 h in TBST buffer (20 mM Tris-Cl, 150 mM NaCl, 0.1% Tween 20, pH 7.4). After blocking, the membranes were probed with primary antibodies, including IL-6 (ABclonal, Woburn), MCP-1 (ABclonal, Woburn), TGF-β (Arigo Laboratories, Hsinchu, Taiwan), and β-actin (Abcam, London, UK) overnight. The desired protein bands were identified using horseradish peroxidase-conjugated secondary antibodies and developed with an enhanced chemiluminescence solution (ThermoFisher, Waltham, MA, USA). Immunoblot imaging was performed using a BioSpectrum AC Imaging System (Ultra-Violet Product Ltd., Cambridge, UK) to capture the protein band signals of immunoreactivity. The protein bands were quantified using ImageQuant software (GE-Healthcare, Marlborough, MA, USA) and digitally converted for statistical analysis.

### 4.12. Statistical Analysis

All data were expressed as mean ± standard error of the mean. Serum parameters and urinary ACR were expressed as absolute values and absolute changes from the sham group, and effects were statistically calculated using one-way analysis of variance (ANOVA) followed by the Brown–Forsythe test. Relative densities of the protein bands were digitized using a UVP bioimaging system, and differences among the ratios of protein/β-actin were evaluated using one-way ANOVA with Prism 6 (GraphPad, San Diego, CA, USA). Degrees of renal fibrosis in observed fields were evaluated using the Image-Pro Plus software, and ratios of Sirius red-stained areas in observed fields for all experimental groups were calculated by one-way ANOVA.

## 5. Conclusions

In the mice with orthotopic xenografts of LLC1 cells in the left upper lobe of the lung, kidney failure as a paraneoplastic syndrome was noted. The pathologic features in the kidneys were collapse of glomerular tufts and brush border lesions in renal tubules. Informatics analysis suggested that the secretory proteins of lung cancer cells potentiated pathological progression in kidneys. The lung cancer secretory proteins are able to induce renal inflammation and loss of glomerular integrity as the renal cells or primary culture glomeruli were exposed to LLC1 cell-derived conditioned medium.

## Figures and Tables

**Table 1:** Proteins secreted by LLC1 cells related to renal injury.

Gene Name	Protein Name	Signaling Pathway in Renal Pathogenesis	Kidney Disease Association	Ref.
*ANGPL2*	Angiopoietin-related protein 2	TGF-β pathway	Renal fibrosis	[37]
*BGN*	Biglycan	TLR-2/4 signaling	Inflammation	[39,42,43]
*BMP1*	Bone morphogenetic protein1	TGF-β pathway	Renal fibrosis	[38]
*C1r*	Complement C1r	Complement system	Renal fibrosis	[44]
*C1s*	Complement C1s	Complement system	Renal fibrosis	[44]
*DCN*	Decorin	TGF-β pathway	Renal fibrosis	[39,43]
*DPP3*	Dipeptidyl peptidase 3	Renin-angiotensin system	Inflammation	[45,46]
*FN1*	Fibronectin	TGF-β pathway	Glomerulopathy, mesangial expansion, renal fibrosis	[40,47]
*LGALS3*	Galectin-3		Interstitial fibrosis, ciliopathy	[48]
*MIF*	Macrophage migration inhibitory factor	TLR-4-NF-β B signaling axis	Acute kidney injury, inflammation	[49]
*MCP1*	Monocyte chemoattractant protein 1	TGF-β pathway	Inflammation, glomerular injury, renal fibrosis	[41,50]
*PDGFC*	Platelet-derived growth factor C	PDGF signaling	Renal interstitial fibrosis	[51,52,53]

## Supplementary Materials

The following are available online at https://www.mdpi.com/2072-6694/12/12/3561/s1. Figure S1: Lung anatomy of the mice that developed lung tumors after LLC1 cell implantation, Figure S2: The lung specimens stained with hematoxylin and eosin. The LLC1 cells grew and formed a tumor, Figure S3: Kidney specimens stained with hematoxylin, Figure S4: Glomerular damage present in the kidneys of the mice with lung cancer, Figure S5: Whole gel figures, Table S1: Physiological parameters of experimental mice, Table S2: Secretory proteins identified in A549 lung cancer cell secretome, Table S3: Secretome profiling of LLC1 cells, Table S4: Secretome profiling of A549 cells, Table S5: List of materials used in the study.

## References

[1] Gao C.F., Xie Q., Su Y.L., Koeman J., Khoo S.K., Gustafson M., Knudsen B.S., Hay R., Shinomiya N., Vande Woude G.F. (2005). Proliferation and invasion: Plasticity in tumor cells. Proc. Natl. Acad. Sci. USA.

[2] Hamilton G., Moser D., Hochmair M. (2016). Metastasis: Circulating Tumor Cells in Small Cell Lung Cancer. Trends Cancer.

[3] Friedl P., Alexander S. (2011). Cancer invasion and the microenvironment: Plasticity and reciprocity. Cell.

[4] Kanaji N., Watanabe N., Kita N., Bandoh S., Tadokoro A., Ishii T., Dobashi H., Matsunaga T. (2014). Paraneoplastic syndromes associated with lung cancer. World. J. Clin. Oncol..

[5] Anwar A., Jafri F., Ashraf S., Jafri M.A.S., Fanucchi M. (2019). Paraneoplastic syndromes in lung cancer and their management. Ann. Transl. Med..

[6] Herbst R.S., Heymach J.V., Lippman S.M. (2008). Lung cancer. N. Engl. J. Med..

[7] Wilson F.P., Berns J.S. (2014). Tumor lysis syndrome: New challenges and recent advances. Adv. Chronic Kidney Dis..

[8] Wilson F.P., Berns J.S. (2012). Onco-nephrology: Tumor lysis syndrome. Clin. J. Am. Soc. Nephrol..

[9] Gandhi L., Johnson B.E. (2006). Paraneoplastic syndromes associated with small cell lung cancer. J. Natl. Compr. Canc. Netw..

[10] Rosner M.H., Perazella M.A. (2017). Acute Kidney Injury in Patients with Cancer. N. Engl. J. Med..

[11] de la Monte S.M., Hutchins G.M., Moore G.W. (1984). Paraneoplastic syndromes and constitutional symptoms in prediction of metastatic behavior of small cell carcinoma of the lung. Am. J. Med..

[12] Henry K. (2019). Paraneoplastic syndromes: Definitions, classification, pathophysiology and principles of treatment. Semin. Diagn. Pathol..

[13] Graus F., Dalmau J. (2019). Paraneoplastic neurological syndromes in the era of immune-checkpoint inhibitors. Nat. Rev. Clin. Oncol..

[14] Wu C.C., Hsu C.W., Chen C.D., Yu C.J., Chang K.P., Tai D.I., Liu H.P., Su W.H., Chang Y.S., Yu J.S. (2010). Candidate serological biomarkers for cancer identified from the secretomes of 23 cancer cell lines and the human protein atlas. Mol. Cell. Proteom..

[15] Yoneda T., Alsina M.A., Chavez J.B., Bonewald L., Nishimura R., Mundy G.R. (1991). Evidence that tumor necrosis factor plays a pathogenetic role in the paraneoplastic syndromes of cachexia, hypercalcemia, and leukocytosis in a human tumor in nude mice. J. Clin. Investig..

[16] Yoneda T., Aufdemorte T.B., Nishimura R., Nishikawa N., Sakuda M., Alsina M.M., Chavez J.B., Mundy G.R. (1991). Occurrence of hypercalcemia and leukocytosis with cachexia in a human squamous cell carcinoma of the maxilla in athymic nude mice: A novel experimental model of three concomitant paraneoplastic syndromes. J. Clin. Oncol..

[17] Ohara G., Satoh H., Kurishima K., Ohtsuka M., Hizawa N. (2009). Paraneoplastic nephrotic syndrome in patients with lung cancer. Intern. Med..

[18] Boon E.S., Vrij A.A., Nieuwhof C., van Noord J.A., Zeppenfeldt E. (1994). Small cell lung cancer with paraneoplastic nephrotic syndrome. Eur. Respir. J..

[19] Herbst R.S., Morgensztern D., Boshoff C. (2018). The biology and management of non-small cell lung cancer. Nature.

[20] Lien Y.H., Lai L.W. (2011). Pathogenesis, diagnosis and management of paraneoplastic glomerulonephritis. Nat. Rev. Nephrol..

[21] Ronco P.M. (1999). Paraneoplastic glomerulopathies: New insights into an old entity. Kidney Int..

[22] Pascal R.R., Slovin S.F. (1980). Tumor directed antibody and carcinoembryonic antigen in the glomeruli of a patient with gastric carcinoma. Hum. Pathol..

[23] Sardhara J., Shukla M., Jamdar J., Jaiswal A.K., Jaiswal S., Kaul A., Bhaisora K.S., Das K.K., Mehrotra A., Behari S. (2018). Paraneoplastic Nephrotic Syndrome in a Patient with Planum Sphenoidale Meningioma. Asian J. Neurosurg..

[24] Campbell G.A., Hu D., Okusa M.D. (2014). Acute kidney injury in the cancer patient. Adv. Chronic Kidney Dis..

[25] Guabello G., Brunetti L., Palladini G., Musumeci S., Lovati E., Perfetti V. (2008). Paraneoplastic Cushing’s syndrome and nephrotic syndrome in a patient with disseminated small cell lung cancer. Am. J. Clin. Oncol..

[26] Antonov A.S., Antonova G.N., Munn D.H., Mivechi N., Lucas R., Catravas J.D., Verin A.D. (2011). alphaVbeta3 integrin regulates macrophage inflammatory responses via PI3 kinase/Akt-dependent NF-kappaB activation. J. Cell. Physiol..

[27] Kassiotis G., Kollias G. (2001). TNF and receptors in organ-specific autoimmune disease: Multi-layered functioning mirrored in animal models. J. Clin. Investig..

[28] Aytekin A., Ozet A., Bilgetekin I., Ogut B., Ciltas A., Benekli M. (2017). A case of membranous glomerulopathy associated with lung cancer and review of the literature. Mol. Clin. Oncol..

[29] Plaisier E., Ronco P. (2020). Screening for Cancer in Patients with Glomerular Diseases. Clin. J. Am. Soc. Nephrol..

[30] Lin F.C., Chen J.Y., Yang A.H., Chang S.C. (2002). The association of non-small-cell lung cancer, focal segmental glomerulosclerosis, and platelet dysfunction. Am. J. Med. Sci..

[31] Niewczas M.A., Pavkov M.E., Skupien J., Smiles A., Md Dom Z.I., Wilson J.M., Park J., Nair V., Schlafly A., Saulnier P.J. (2019). A signature of circulating inflammatory proteins and development of end-stage renal disease in diabetes. Nat. Med..

[32] Bacchetta J., Juillard L., Cochat P., Droz J.P. (2009). Paraneoplastic glomerular diseases and malignancies. Crit. Rev. Oncol. Hematol..

[33] Feng L., Yang Y., Li M., Song J., Gao Y., Cheng S., Xiao T. (2018). Systems biology analysis of the lung cancerrelated secretome. Oncol. Rep..

[34] Shin J., Song S.Y., Ahn H.S., An B.C., Choi Y.D., Yang E.G., Na K.J., Lee S.T., Park J.I., Kim S.Y. (2017). Integrative analysis for the discovery of lung cancer serological markers and validation by MRM-MS. PLoS ONE.

[35] Baradaran A., Nasri H. (2014). Comment on: Clinical significance of IgG deposition in the glomerular mesangial area in patients with IgA nephropathy. Clin. Exp. Nephrol..

[36] Wada Y., Ogata H., Takeshige Y., Takeshima A., Yoshida N., Yamamoto M., Ito H., Kinugasa E. (2013). Clinical significance of IgG deposition in the glomerular mesangial area in patients with IgA nephropathy. Clin. Exp. Nephrol..

[37] Ishii T., Furuya F., Takahashi K., Shikata M., Takamura T., Kobayashi H., Miyazaki A., Morinaga J., Terada K., Oike Y. (2019). Angiopoietin-Like Protein 2 Promotes the Progression of Diabetic Kidney Disease. J. Clin. Endocrinol. Metab..

[38] Grgurevic L., Macek B., Healy D.R., Brault A.L., Erjavec I., Cipcic A., Grgurevic I., Rogic D., Galesic K., Brkljacic J. (2011). Circulating bone morphogenetic protein 1–3 isoform increases renal fibrosis. J. Am. Soc. Nephrol..

[39] Markmann A., Hausser H., Schonherr E., Kresse H. (2000). Influence of decorin expression on transforming growth factor-beta-mediated collagen gel retraction and biglycan induction. Matrix Biol..

[40] Klemis V., Ghura H., Federico G., Wurfel C., Bentmann A., Gretz N., Miyazaki T., Grone H.J., Nakchbandi I.A. (2017). Circulating fibronectin contributes to mesangial expansion in a murine model of type 1 diabetes. Kidney Int..

[41] Eardley K.S., Zehnder D., Quinkler M., Lepenies J., Bates R.L., Savage C.O., Howie A.J., Adu D., Cockwell P. (2006). The relationship between albuminuria, MCP-1/CCL2, and interstitial macrophages in chronic kidney disease. Kidney Int..

[42] Poluzzi C., Nastase M.V., Zeng-Brouwers J., Roedig H., Hsieh L.T., Michaelis J.B., Buhl E.M., Rezende F., Manavski Y., Bleich A. (2019). Biglycan evokes autophagy in macrophages via a novel CD44/Toll-like receptor 4 signaling axis in ischemia/reperfusion injury. Kidney Int..

[43] Stokes M.B., Holler S., Cui Y., Hudkins K.L., Eitner F., Fogo A., Alpers C.E. (2000). Expression of decorin, biglycan, and collagen type I in human renal fibrosing disease. Kidney Int..

[44] Lesher A.M., Song W.C. (2010). Review: Complement and its regulatory proteins in kidney diseases. Nephrology (Carlton).

[45] Depret F., Amzallag J., Pollina A., Fayolle-Pivot L., Coutrot M., Chaussard M., Santos K., Hartmann O., Jully M., Fratani A. (2020). Circulating dipeptidyl peptidase-3 at admission is associated with circulatory failure, acute kidney injury and death in severely ill burn patients. Crit. Care.

[46] Jha S., Taschler U., Domenig O., Poglitsch M., Bourgeois B., Pollheimer M., Pusch L.M., Malovan G., Frank S., Madl T. (2020). Dipeptidyl peptidase 3 modulates the renin-angiotensin system in mice. J. Biol. Chem..

[47] Lusco M.A., Chen Y.P., Cheng H., Dong H.R., Najafian B., Alpers C.E., Fogo A.B. (2017). AJKD Atlas of Renal Pathology: Fibronectin Glomerulopathy. Am. J. Kidney. Dis..

[48] Chen S.C., Kuo P.L. (2016). The Role of Galectin-3 in the Kidneys. Int. J. Mol. Sci..

[49] Li J.H., Tang Y., Lv J., Wang X.H., Yang H., Tang P.M.K., Huang X.R., He Z.J., Zhou Z.J., Huang Q.Y. (2019). Macrophage migration inhibitory factor promotes renal injury induced by ischemic reperfusion. J. Cell. Mol. Med..

[50] Tesch G.H., Schwarting A., Kinoshita K., Lan H.Y., Rollins B.J., Kelley V.R. (1999). Monocyte chemoattractant protein-1 promotes macrophage-mediated tubular injury, but not glomerular injury, in nephrotoxic serum nephritis. J. Clin. Investig..

[51] van Roeyen C.R.C., Martin I.V., Drescher A., Schuett K.A., Hermert D., Raffetseder U., Otten S., Buhl E.M., Braun G.S., Kuppe C. (2019). Identification of platelet-derived growth factor C as a mediator of both renal fibrosis and hypertension. Kidney Int..

[52] Floege J., Eitner F., Alpers C.E. (2008). A new look at platelet-derived growth factor in renal disease. J. Am. Soc. Nephrol..

[53] Eitner F., Bucher E., van Roeyen C., Kunter U., Rong S., Seikrit C., Villa L., Boor P., Fredriksson L., Backstrom G. (2008). PDGF-C is a proinflammatory cytokine that mediates renal interstitial fibrosis. J. Am. Soc. Nephrol..

[54] Choudhary N., Ahlawat R.S. (2008). Interleukin-6 and C-reactive protein in pathogenesis of diabetic nephropathy: New evidence linking inflammation, glycemic control, and microalbuminuria. Iran J. Kidney Dis..

[55] Lee S.J., Borsting E., Decleves A.E., Singh P., Cunard R. (2012). Podocytes express IL-6 and lipocalin 2/neutrophil gelatinase-associated lipocalin in lipopolysaccharide-induced acute glomerular injury. Nephron Exp. Nephrol..

[56] Borthwick L.A., Wynn T.A., Fisher A.J. (2013). Cytokine mediated tissue fibrosis. Biochim. Biophys. Acta.

[57] Eddy A.A. (2014). Overview of the cellular and molecular basis of kidney fibrosis. Kidney Int. Suppl..

[58] Blaine J., Dylewski J. (2020). Regulation of the Actin Cytoskeleton in Podocytes. Cells.

[59] Zhang G., Liu Z., Ding H., Miao H., Garcia J.M., Li Y.P. (2017). Toll-like receptor 4 mediates Lewis lung carcinoma-induced muscle wasting via coordinate activation of protein degradation pathways. Sci. Rep..

[60] Zager R.A. (1996). Rhabdomyolysis and myohemoglobinuric acute renal failure. Kidney Int..

[61] Belliere J., Casemayou A., Ducasse L., Zakaroff-Girard A., Martins F., Iacovoni J.S., Guilbeau-Frugier C., Buffin-Meyer B., Pipy B., Chauveau D. (2015). Specific macrophage subtypes influence the progression of rhabdomyolysis-induced kidney injury. J. Am. Soc. Nephrol..

[62] Nasr S.H., Fogo A.B. (2019). New developments in the diagnosis of fibrillary glomerulonephritis. Kidney Int..

[63] Rops A., Jansen E., van der Schaaf A., Pieterse E., Rother N., Hofstra J., Dijkman H., van de Logt A.E., Wetzels J., van der Vlag J. (2018). Interleukin-6 is essential for glomerular immunoglobulin A deposition and the development of renal pathology in Cd37-deficient mice. Kidney Int..

[64] Rosales I.A., Colvin R.B. (2016). Glomerular disease with idiopathic linear immunoglobulin deposition: A rose by any other name would be atypical. Kidney Int..

[65] Selvaskandan H., Cheung C.K., Muto M., Barratt J. (2019). New strategies and perspectives on managing IgA nephropathy. Clin. Exp. Nephrol..

[66] Devasahayam J., Erode-Singaravelu G., Bhat Z., Oliver T., Chandran A., Zeng X., Dakshinesh P., Pillai U. (2015). C1q Nephropathy: The Unique Underrecognized Pathological Entity. Anal. Cell. Pathol..

[67] Xavier S., Sahu R.K., Bontha S.V., Mass V., Taylor R.P., Megyesi J., Thielens N.M., Portilla D. (2019). Complement C1r serine protease contributes to kidney fibrosis. Am. J. Physiol. Renal Physiol..

[68] Boudhabhay I., Poillerat V., Grunenwald A., Torset C., Leon J., Daugan M.V., Lucibello F., El Karoui K., Ydee A., Chauvet S. (2020). Complement activation is a crucial driver of acute kidney injury in rhabdomyolysis. Kidney Int..

[69] Cosio F.G., Bakaletz A.P. (1984). Abnormal plasma fibronectin levels in patients with proteinuria. J. Lab. Clin. Med..

[70] Jhaveri K.D., Shah H.H., Patel C., Kadiyala A., Stokes M.B., Radhakrishnan J. (2014). Glomerular diseases associated with cancer, chemotherapy, and hematopoietic stem cell transplantation. Adv. Chronic Kidney Dis..

