# Supplementary material for: Lung Cancer Cell-Derived Secretome Mediates Paraneoplastic Inflammation and Fibrosis in Kidney in Mice
Source: Cancers (Basel). 2020 Nov 28;12(12):3561. doi: 10.3390/cancers12123561 (PMC7760555; doi:10.3390/cancers12123561)
Supplement: Supplementary file 1 [file cancers-12-03561-s001.pdf]

Supplementary Materials: Lung Cancer Cell-Derived Secretome Mediates Paraneoplastic Inflammation and Fibrosis in Kidney in Mice

Chi-Chih Hung, Yen-Yi Zhen, Sheng-Wen Niu, Jui-Feng Hsu, Tai-Huang Lee, Hsiang-Hao Chuang, Pei-Hui Wang, Su-Chu Lee, Pi-Chen Lin, Yi-Wen Chiu, Chien-Hsing Wu, Ming-Shyan Huang, Michael Hsiao, Hung-Chun Chen and Chih-Jen Yang

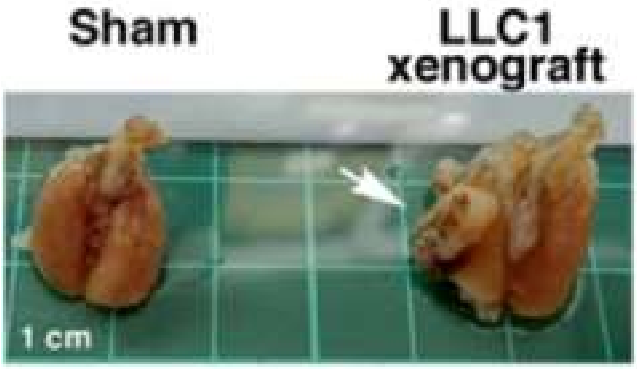

Figure S1. Anatomical view of lung cancer in the left upper lobe of lung. The arrow points position of the lung tumor.

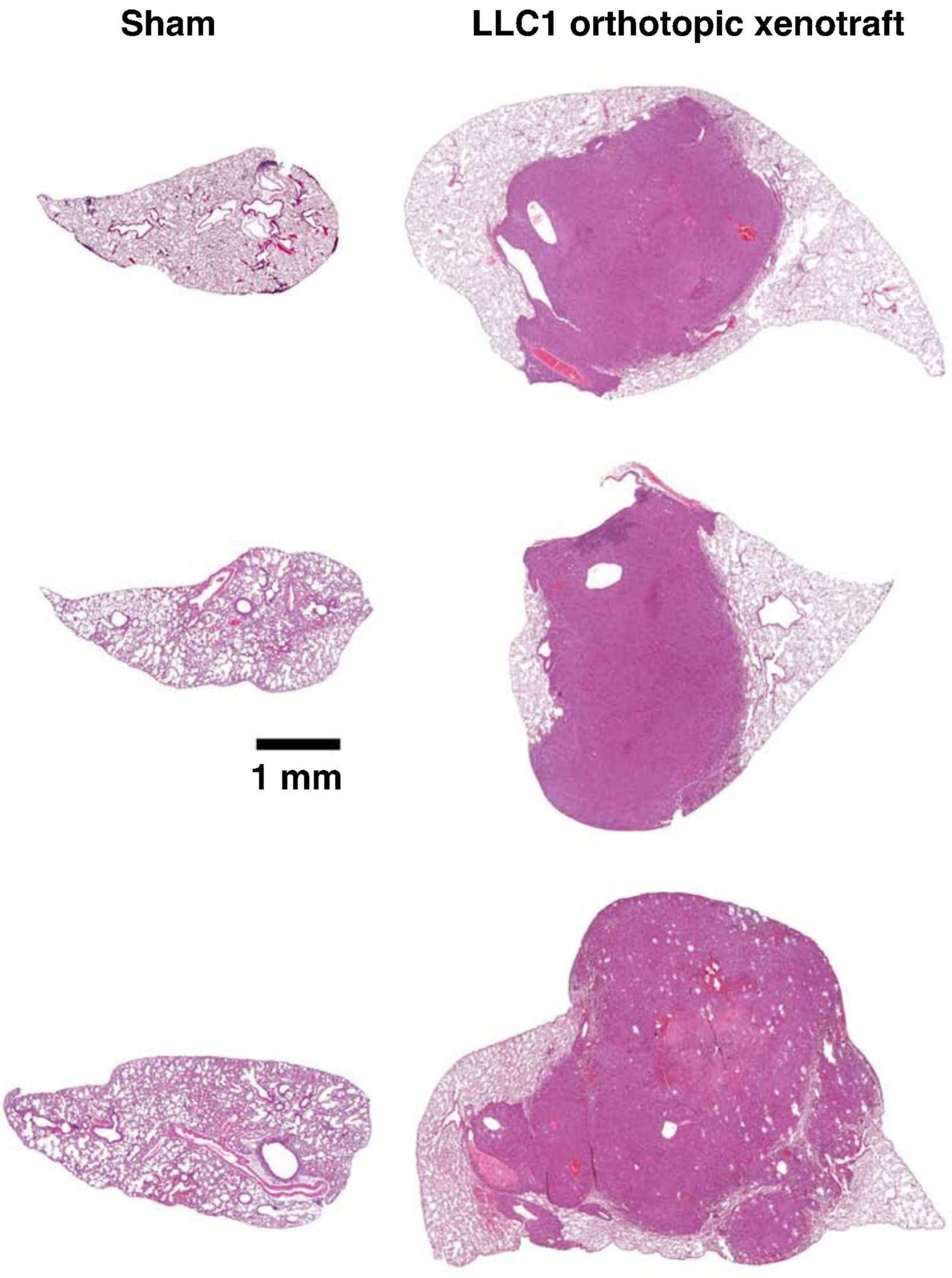

Figure S2. Hematoxylin-eosin stained upper lobe of lung showed massive lung cancer in cross-section of lung.

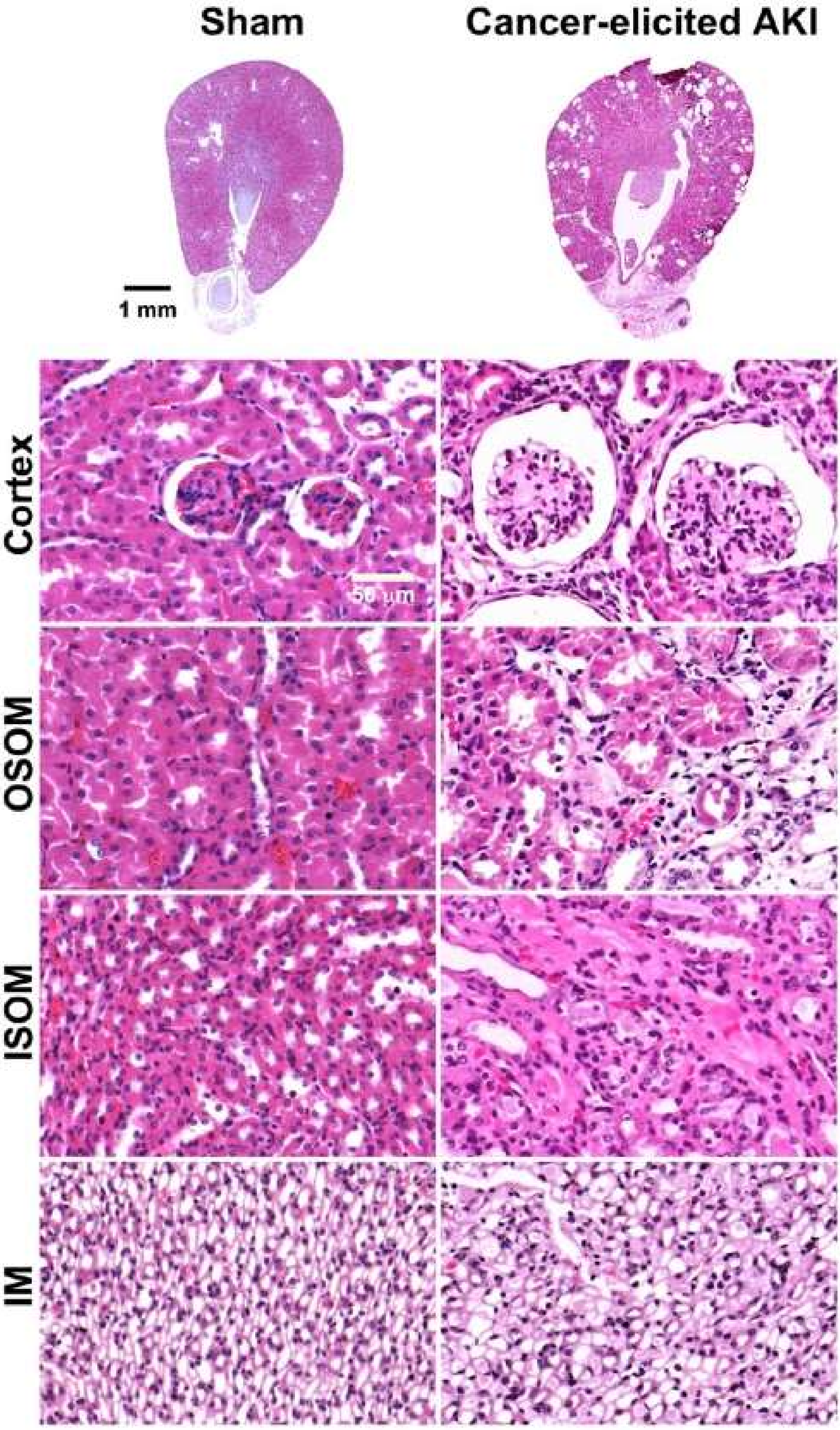

Figure S3. Kidney histopathology were stained with Hematoxylin and eosin. Glomerular lesions are clearly visualized in the biopsy from lung cancer mice.

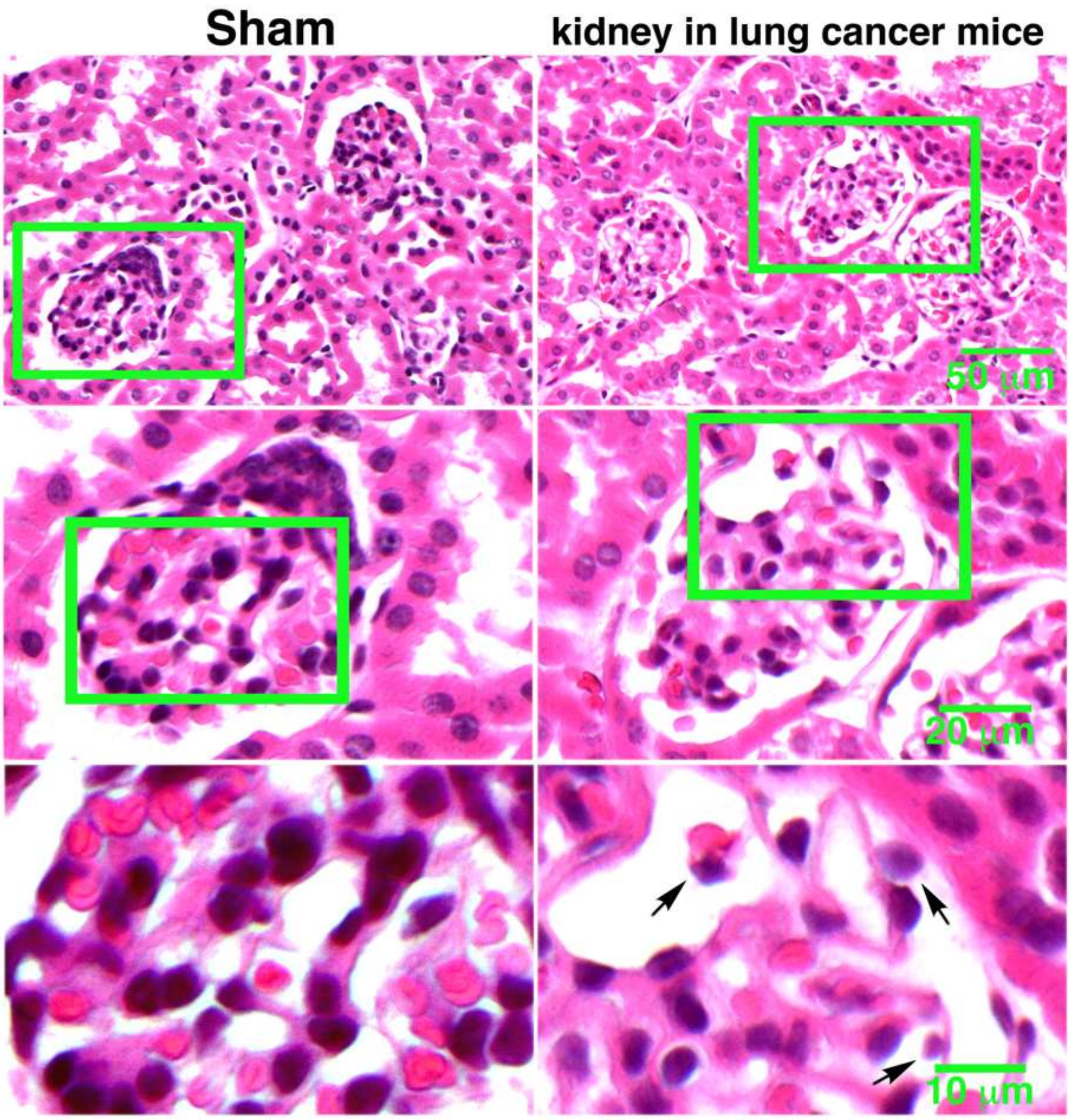

Figure S4. AT higher magnification as detailed glomerular morphology showed capillary dilation and cells in lumen of capillary, as noted the arrow pointed positions.

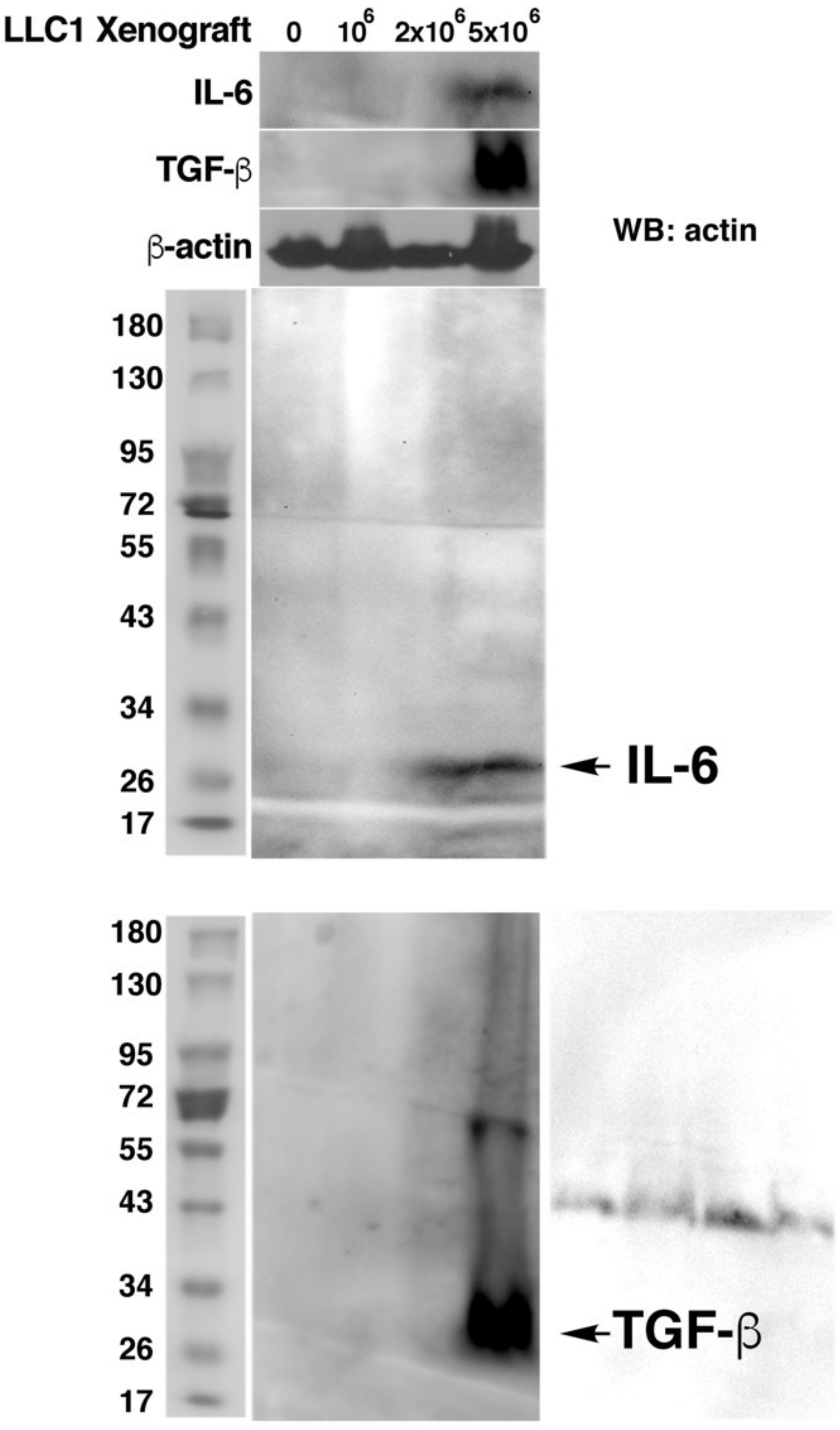

Figure S5. *Cont.*

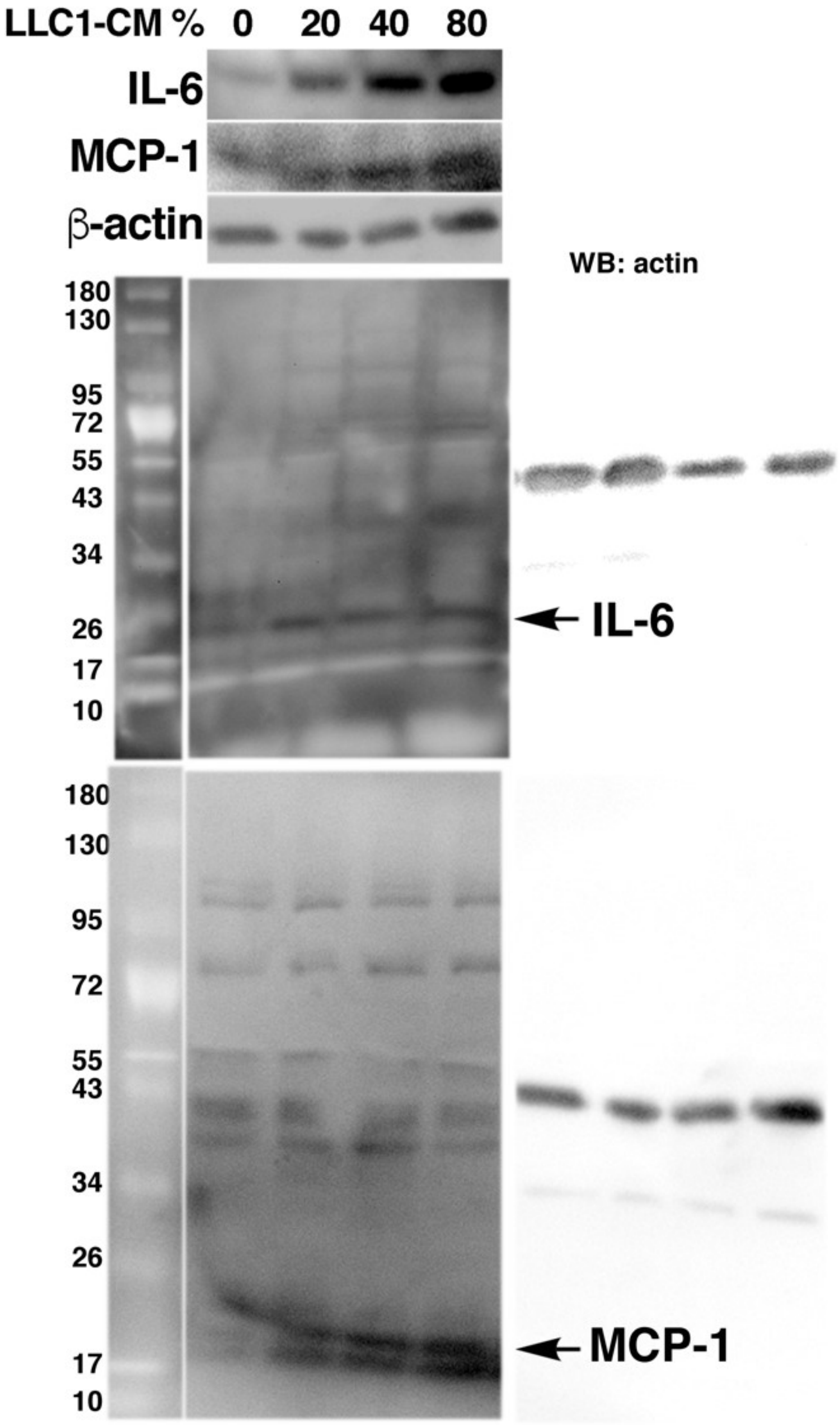

Figure S5. Whole gel pictures.

Table S1. Physiological and pathological Parameters in experiment mice.

Exp. Group	Animal Use Number	Survival Animal number	Body Weight Loss %	Tumor Size (%): Tumor Area in Lung cross-section	ACR (μg/mg)	Mouse Activity
**Sham**	4	4	-1.23±0.645	0	33.87±2.151	normal
**1 × 10⁶**	6	4	5.525±1.028		220.6±71.64	lower
**2 × 10⁶**	6	4	13.53±1.149		239.8±95.59	lower
**5 × 10⁶**	7	5	20.45±1.728	66.22±77.41	803.7±255.3	lower

Table S2. Secretory proteins identified in A549 lung cancer cell secretome.

Gene Name	Protein Name	Signaling Pathway in Renal Pathogenesis	Role in Kidney Disease	Ref
**C1r**	Complement C1s	Complement system	Renal fibrosis	
**C1s**	Complement C1s	Complement system	Renal fibrosis	
CXCL1	C-X-C motif chemokine ligand 1		AKI, Inflammation	[1]
CXCL3	C-X-C motif chemokine ligand 3	TGF- pathway	Renal fibrosis	[2]
CXCL5	C-X-C motif chemokine ligand 5		Kidney cancer	[3]
CXCL8	C-X-C motif chemokine ligand 8		Renal inflammation, Renal allograft damage	[4]
MCP1/CCL2	C-C motif chemokine ligand 2		AKI, Renal inflammatory disease	[5]
SPP1	secreted phosphoprotein 1		AKI, CKD, renal allograft dysfunction	[6]
TGF-β2	transforming growth factor β 2	TGF- pathway	Renal fibrosis	[7]
TGF-βI	transforming growth factor β induced protein	TGF- pathway		
TNFRSF12A	TNF receptor superfamily member 12A	TWEAK-Fn14 pathway	CKD, Renal fibrosis	[8]
TNFRSF1A	TNF receptor superfamily member 1A	TNF-α-TNFRSF1A pathway	Glomerulosclerosis, interstitial fibrosis	[9]

Table S3. List of LLC1 cells secretome profiling.

Symbol	Entrez Gene Name	GenPept	Location
AARS1	*alanyl-tRNA synthetase 1*	Q8BGQ7	Cytoplasm
ABRACL	*ABRA C-terminal like*	Q4KML4	Other
ACOT7	*acyl-CoA thioesterase 7*	Q91V12	Cytoplasm
ACP1	*acid phosphatase 1*	Q9D358	Cytoplasm
ACTB	*actin beta*	P60710	Cytoplasm
ACTBL2	*actin beta like 2*	Q8BFZ3	Nucleus
ACTC1	*actin alpha cardiac muscle 1*	P68033	Cytoplasm
ACTL6A	*actin like 6A*	Q9Z2N8	Nucleus
ACTN1	*actinin alpha 1*	Q7TPR4	Cytoplasm
ACTN4	*actinin alpha 4*	P57780	Cytoplasm
ACTR2	*actin related protein 2*	P61161	Plasma Membrane
ACTR3	*actin related protein 3*	Q99JY9	Plasma Membrane
ADPRH	*ADP-ribosylarginine hydrolase*	P54923	Other
ADSL	*adenylosuccinate lyase*	P54822	Cytoplasm
ADSS1	*adenylosuccinate synthase 1*	P28650	Cytoplasm
ADSS2	*adenylosuccinate synthase 2*	P46664	Cytoplasm
AEBP1	*AE binding protein 1*	Q640N1	Nucleus
AGFG1	*ArfGAP with FG repeats 1*	Q8K2K6	Nucleus
AHCY	*adenosylhomocysteinase*	P50247	Cytoplasm
AHSA1	*activator of HSP90 ATPase activity 1*	Q8BK64	Cytoplasm
AIMP1	*aminoacyl tRNA synthetase complex interacting multifunctional protein 1*	P31230	Extracellular Space
AK1	*adenylate kinase 1*	Q9R0Y5	Cytoplasm
AKR1A1	*aldo-keto reductase family 1 member A1*	Q9JII6	Cytoplasm
AKR1B1	*aldo-keto reductase family 1 member B*	P45376	Cytoplasm
AKR1B10	*aldo-keto reductase family 1 member B10*	P45377	Cytoplasm
ALB	*albumin*	P07724	Extracellular Space
ALDOA	*aldolase, fructose-bisphosphate A*	P05064	Cytoplasm
ANGPTL2	*angiopoietin like 2*	Q9R045	Extracellular Space
Anp32a	*acidic (leucine-rich) nuclear phosphoprotein 32 family, member A*	O35381	Nucleus
Anp32b	*acidic (leucine-rich) nuclear phosphoprotein 32 family, member B*	Q9EST5	Nucleus
Anp32e	*acidic (leucine-rich) nuclear phosphoprotein 32 family, member E*	P97822	Cytoplasm
ANXA1	*annexin A1*	P10107	Plasma Membrane
ANXA2	*annexin A2*	P07356	Plasma Membrane

ANXA3	*annexin A3*	O35639	Cytoplasm
ANXA5	*annexin A5*	P48036	Plasma Membrane
APCDD1	*APC down-regulated 1*	Q3U128	Plasma Membrane
APEX1	*apurinic/apyrimidinic endodeoxyribonuclease 1*	P28352	Nucleus
API5	*apoptosis inhibitor 5*	O35841	Cytoplasm
APLP2	*amyloid beta precursor like protein 2*	Q06335	Cytoplasm
APRT	*adenine phosphoribosyltransferase*	P08030	Cytoplasm
ARCN1	*archain 1*	Q5XJY5	Cytoplasm
ARF3	*ADP ribosylation factor 3*	P61205	Cytoplasm
ARHGDIA	*Rho GDP dissociation inhibitor alpha*	Q99PT1	Cytoplasm
ARPC2	*actin related protein 2/3 complex subunit 2*	Q9CVB6	Cytoplasm
ARPC4	*actin related protein 2/3 complex subunit 4*	P59999	Cytoplasm
ASNS	*asparagine synthetase (glutamine-hydrolyzing)*	Q61024	Cytoplasm
ATIC	*5-aminoimidazole-4-carboxamide ribonucleotide formyltransferase/IMP cyclohydrolase*	Q9CWJ9	Cytoplasm
ATOX1	*antioxidant 1 copper chaperone*	O08997	Cytoplasm
ATP6AP1	*ATPase H+ transporting accessory protein 1*	Q9R1Q9	Cytoplasm
ATXN10	*ataxin 10*	P28658	Cytoplasm
AXL	*AXL receptor tyrosine kinase*	Q00993	Plasma Membrane
B2M	*beta-2-microglobulin*	P01887	Plasma Membrane
B4GALT1	*beta-1,4-galactosyltransferase 1*	P15535	Cytoplasm
BGN	*biglycan*	P28653	Extracellular Space
BIN1	*bridging integrator 1*	O08539	Nucleus
BLVRB	*biliverdin reductase B*	Q923D2	Cytoplasm
BMP1	*bone morphogenetic protein 1*	P98063	Extracellular Space
BUB3	*BUB3 mitotic checkpoint protein*	Q9WVA3	Nucleus
BZW2	*basic leucine zipper and W2 domains 2*	Q2L4X1	Cytoplasm
C11orf58	*chromosome 11 open reading frame 58*	Q9R0P4	Other
C1R	*complement C1r*	Q8CFG9	Extracellular Space
C1S	*complement C1s*	Q8CG14	Extracellular Space
CACYBP	*calcyclin binding protein*	Q9CXW3	Nucleus
Calm1 (includes others)	*calmodulin 1*	P0DP27	Nucleus
CALR	*calreticulin*	P14211	Cytoplasm
CALU	*calumenin*	O35887	Cytoplasm
CAND1	*cullin associated and neddylation dissociated 1*	Q6ZQ38	Cytoplasm
CANX	*calnexin*	P35564	Cytoplasm
CAP1	*cyclase associated actin cytoskeleton regulatory protein 1*	P40124	Plasma Membrane

CAPG	*capping actin protein, gelsolin like*	P24452	Nucleus
CAPN2	*calpain 2*	O08529	Cytoplasm
CAPNS1	*calpain small subunit 1*	O88456	Cytoplasm
CAPRIN1	*cell cycle associated protein 1*	Q60865	Plasma Membrane
CAPZA1	*capping actin protein of muscle Z-line subunit alpha 1*	P47753	Cytoplasm
CAPZA2	*capping actin protein of muscle Z-line subunit alpha 2*	P47754	Cytoplasm
CAPZB	*capping actin protein of muscle Z-line subunit beta*	P47757	Cytoplasm
CASP3	*caspase 3*	P70677	Cytoplasm
CAST	*calpastatin*	P51125	Cytoplasm
CAVIN1	*caveolae associated protein 1*	O54724	Nucleus
CAVIN2	*caveolae associated protein 2*	Q63918	Plasma Membrane
CBX3	*chromobox 3*	P23198	Nucleus
Ccl2	*chemokine (C-C motif) ligand 2*	P10148	Extracellular Space
CCN3	*cellular communication network factor 3*	Q64299	Extracellular Space
CCT2	*chaperonin containing TCP1 subunit 2*	P80314	Cytoplasm
CCT3	*chaperonin containing TCP1 subunit 3*	P80318	Cytoplasm
CCT4	*chaperonin containing TCP1 subunit 4*	P80315	Cytoplasm
CCT5	*chaperonin containing TCP1 subunit 5*	P80316	Cytoplasm
CCT7	*chaperonin containing TCP1 subunit 7*	P80313	Cytoplasm
CCT8	*chaperonin containing TCP1 subunit 8*	P42932	Cytoplasm
CCT6A	*chaperonin containing TCP1 subunit 6A*	P80317	Cytoplasm
CD81	*CD81 molecule*	P35762	Plasma Membrane
CD109	*CD109 molecule*	Q8R422	Plasma Membrane
CDC37	*cell division cycle 37, HSP90 cochaperone*	Q61081	Cytoplasm
CDV3	*CDV3 homolog*	Q4VAA2	Cytoplasm
CFDP1	*craniofacial development protein 1*	O88271	Extracellular Space
CFL1	*cofilin 1*	P18760	Nucleus
CLIC1	*chloride intracellular channel 1*	Q9Z1Q5	Nucleus
CLSTN1	*calsyntenin 1*	Q9EPL2	Plasma Membrane
CLTC	*clathrin heavy chain*	Q68FD5	Plasma Membrane
CLU	*clusterin*	Q06890	Cytoplasm
CMPK1	*cytidine/uridine monophosphate kinase 1*	Q9DBP5	Nucleus
CNBP	*CCHC-type zinc finger nucleic acid binding protein*	P53996	Nucleus
CNDP2	*carnosine dipeptidase 2*	Q9D1A2	Cytoplasm
CNN3	*calponin 3*	Q9DAW9	Cytoplasm
COL18A1	*collagen type XVIII alpha 1 chain*	P39061	Extracellular Space

COL3A1	*collagen type III alpha 1 chain*	P08121	Extracellular Space
COL4A1	*collagen type IV alpha 1 chain*	P02463	Extracellular Space
COL4A2	*collagen type IV alpha 2 chain*	P08122	Extracellular Space
COL5A1	*collagen type V alpha 1 chain*	O88207	Extracellular Space
COL6A1	*collagen type VI alpha 1 chain*	Q04857	Extracellular Space
COPB1	*COPI coat complex subunit beta 1*	Q9JIF7	Cytoplasm
COPB2	*COPI coat complex subunit beta 2*	O55029	Cytoplasm
COPE	*COPI coat complex subunit epsilon*	O89079	Cytoplasm
CORO1B	*coronin 1B*	Q9WUM3	Cytoplasm
COX6B1	*cytochrome c oxidase subunit 6B1*	P56391	Cytoplasm
CP	*ceruloplasmin*	Q61147	Extracellular Space
CPE	*carboxypeptidase E*	Q00493	Cytoplasm
CPQ	*carboxypeptidase Q*	Q9WVJ3	Extracellular Space
CPSF6	*cleavage and polyadenylation specific factor 6*	Q6NVF9	Nucleus
CRABP1	*cellular retinoic acid binding protein 1*	P62965	Cytoplasm
CRK	*CRK proto-oncogene, adaptor protein*	Q64010	Cytoplasm
CSE1L	*chromosome segregation 1 like*	Q9ERK4	Nucleus
CSF1	*colony stimulating factor 1*	P07141	Extracellular Space
CST3	*cystatin C*	P21460	Extracellular Space
CSTB	*cystatin B*	Q62426	Cytoplasm
CTBS	*chitobiase*	Q8R242	Cytoplasm
CTSB	*cathepsin B*	P10605	Cytoplasm
CTSV	*cathepsin V*	P06797	Cytoplasm
CYCS	*cytochrome c, somatic*	P62897	Cytoplasm
CYRIB	*CYFIP related Rac1 interactor B*	Q921M7	Extracellular Space
DARS1	*aspartyl-tRNA synthetase 1*	Q922B2	Cytoplasm
DBI	*diazepam binding inhibitor, acyl-CoA binding protein*	P31786	Cytoplasm
DCN	*decorin*	P28654	Extracellular Space
DCPS	*decapping enzyme, scavenger*	Q9DAR7	Nucleus
DDB1	*damage specific DNA binding protein 1*	Q3U1J4	Nucleus
DDX17	*DEAD-box helicase 17*	Q501J6	Nucleus
DDX21	*DExD-box helicase 21*	Q9JIK5	Nucleus
DDX39B	*DExD-box helicase 39B*	Q9Z1N5	Nucleus
DEK	*DEK proto-oncogene*	Q7TNV0	Nucleus
DHX9	*DExH-box helicase 9*	O70133	Nucleus
DPP3	*dipeptidyl peptidase 3*	Q99KK7	Cytoplasm

DPYSL2	*dihydropyrimidinase like 2*	O08553	Cytoplasm
DPYSL3	*dihydropyrimidinase like 3*	Q62188	Cytoplasm
DSTN	*destrin, actin depolymerizing factor*	Q9R0P5	Cytoplasm
DYNC1H1	*dynein cytoplasmic 1 heavy chain 1*	Q9JHU4	Cytoplasm
DYNLL1	*dynein light chain LC8-type 1*	P63168	Cytoplasm
ECM1	*extracellular matrix protein 1*	Q61508	Extracellular Space
EDF1	*endothelial differentiation related factor 1*	Q9JMG1	Nucleus
EEF2	*eukaryotic translation elongation factor 2*	P58252	Cytoplasm
EEF1A1	*eukaryotic translation elongation factor 1 alpha 1*	P10126	Cytoplasm
EEF1B2	*eukaryotic translation elongation factor 1 beta 2*	O70251	Cytoplasm
EEF1D	*eukaryotic translation elongation factor 1 delta*	P57776	Cytoplasm
EEF1G	*eukaryotic translation elongation factor 1 gamma*	Q9D8N0	Cytoplasm
EFEMP1	*EGF containing fibulin extracellular matrix protein 1*	Q8BPB5	Extracellular Space
EFEMP2	*EGF containing fibulin extracellular matrix protein 2*	Q9WVJ9	Extracellular Space
EFHD2	*EF-hand domain family member D2*	Q9D8Y0	Other
Eif1	*eukaryotic translation initiation factor 1*	P48024	Other
EIF5	*eukaryotic translation initiation factor 5*	P59325	Cytoplasm
EIF6	*eukaryotic translation initiation factor 6*	O55135	Cytoplasm
EIF2S1	*eukaryotic translation initiation factor 2 subunit alpha*	Q6ZWX6	Cytoplasm
EIF2S2	*eukaryotic translation initiation factor 2 subunit beta*	Q99L45	Cytoplasm
EIF2S3	*eukaryotic translation initiation factor 2 subunit gamma*	Q9Z0N1	Cytoplasm
EIF3A	*eukaryotic translation initiation factor 3 subunit A*	P23116	Cytoplasm
EIF3B	*eukaryotic translation initiation factor 3 subunit B*	Q8JZQ9	Cytoplasm
EIF3E	*eukaryotic translation initiation factor 3 subunit E*	P60229	Cytoplasm
EIF3G	*eukaryotic translation initiation factor 3 subunit G*	Q9Z1D1	Cytoplasm
EIF3J	*eukaryotic translation initiation factor 3 subunit J*	Q3UGC7	Cytoplasm
EIF3M	*eukaryotic translation initiation factor 3 subunit M*	Q99JX4	Cytoplasm
EIF4A1	*eukaryotic translation initiation factor 4A1*	P60843	Cytoplasm
EIF4B	*eukaryotic translation initiation factor 4B*	Q8BGD9	Cytoplasm
EIF4E	*eukaryotic translation initiation factor 4E*	P63073	Cytoplasm
EIF4G1	*eukaryotic translation initiation factor 4 gamma 1*	Q6NZJ6	Cytoplasm
EIF5A2	*eukaryotic translation initiation factor 5A2*	Q8BGY2	Cytoplasm
ELAVL1	*ELAV like RNA binding protein 1*	P70372	Cytoplasm
ELOC	*elongin C*	P83940	Nucleus
EMILIN1	*elastin microfibril interfacer 1*	Q99K41	Extracellular Space
ENO1	*enolase 1*	P17182	Cytoplasm

ENPP2	*ectonucleotide pyrophosphatase/phosphodiesterase 2*	Q9R1E6	Plasma Membrane
EPRS1	*glutamyl-prolyl-tRNA synthetase 1*	Q8CGC7	Cytoplasm
ERH	*ERH mRNA splicing and mitosis factor*	P84089	Nucleus
ESD	*esterase D*	Q9R0P3	Cytoplasm
ETF1	*eukaryotic translation termination factor 1*	Q8BWY3	Cytoplasm
EZR	*ezrin*	P26040	Plasma Membrane
FAM3C	*FAM3 metabolism regulating signaling molecule C*	Q91VU0	Extracellular Space
FASN	*fatty acid synthase*	P19096	Cytoplasm
FKBP3	*FKBP prolyl isomerase 3*	Q62446	Nucleus
FKBP4	*FKBP prolyl isomerase 4*	P30416	Nucleus
FKBP1A	*FKBP prolyl isomerase 1A*	P26883	Cytoplasm
FLNA	*filamin A*	Q8BTM8	Cytoplasm
FLNB	*filamin B*	Q80X90	Cytoplasm
FLNC	*filamin C*	Q8VHX6	Cytoplasm
FN1	*fibronectin 1*	P11276	Extracellular Space
FSCN1	*fascin actin-bundling protein 1*	Q61553	Cytoplasm
FSTL1	*follistatin like 1*	Q62356	Extracellular Space
G3BP1	*G3BP stress granule assembly factor 1*	P97855	Nucleus
GALK1	*galactokinase 1*	Q9R0N0	Cytoplasm
GALNT2	*polypeptide N-acetylgalactosaminyltransferase 2*	Q6PB93	Cytoplasm
GANAB	*glucosidase II alpha subunit*	Q8BHN3	Cytoplasm
GAPDH	*glyceraldehyde-3-phosphate dehydrogenase*	P16858	Cytoplasm
GARS1	*glycyl-tRNA synthetase 1*	Q9CZD3	Cytoplasm
GART	*phosphoribosylglycinamide formyltransferase, phosphoribosylglycinamide synthetase, phosphoribosylaminoimidazole synthetase*	Q64737	Cytoplasm
GCSH	*glycine cleavage system protein H*	Q91WK5	Cytoplasm
GDI1	*GDP dissociation inhibitor 1*	P50396	Cytoplasm
GDI2	*GDP dissociation inhibitor 2*	Q61598	Cytoplasm
GLO1	*glyoxalase I*	Q9CPU0	Cytoplasm
GLOD4	*glyoxalase domain containing 4*	Q9CPV4	Cytoplasm
GLRX3	*glutaredoxin 3*	Q9CQM9	Cytoplasm
Gm21596/Hmgb1	*high mobility group box 1*	P63158	Nucleus
GMFB	*glia maturation factor beta*	Q9CQI3	Cytoplasm
GOT1	*glutamic-oxaloacetic transaminase 1*	P05201	Cytoplasm
GOT2	*glutamic-oxaloacetic transaminase 2*	P05202	Cytoplasm
GPC1	*glypican 1*	Q9QZF2	Plasma Membrane

GPI	*glucose-6-phosphate isomerase*	P06745	Extracellular Space
GRN	*granulin precursor*	P28798	Extracellular Space
GSN	*gelsolin*	P13020	Extracellular Space
GSPT1	*G1 to S phase transition 1*	Q8R050	Cytoplasm
GSTM1	*glutathione S-transferase mu 1*	P15626	Cytoplasm
GSTM5	*glutathione S-transferase mu 5*	P10649	Cytoplasm
GSTO1	*glutathione S-transferase omega 1*	O09131	Cytoplasm
GSTP1	*glutathione S-transferase pi 1*	P19157	Cytoplasm
H1-3	*H1.3 linker histone, cluster member*	P43277	Nucleus
H1f4	*H1.4 linker histone, cluster member*	P43274	Nucleus
HAGH	*hydroxyacylglutathione hydrolase*	Q99KB8	Cytoplasm
HARS1	*histidyl-tRNA synthetase 1*	Q61035	Cytoplasm
HDGF	*heparin binding growth factor*	P51859	Extracellular Space
HINT1	*histidine triad nucleotide binding protein 1*	P70349	Nucleus
HLA-A	*major histocompatibility complex, class I, A*	P01899	Plasma Membrane
Hmga2	*high mobility group AT-hook 2*	P52927	Nucleus
HMGB2	*high mobility group box 2*	P30681	Nucleus
Hnrnpa1	*heterogeneous nuclear ribonucleoprotein A1*	P49312	Nucleus
Hnrnpa3	*heterogeneous nuclear ribonucleoprotein A3*	Q8BG05	Nucleus
HNRNPA2B1	*heterogeneous nuclear ribonucleoprotein A2/B1*	O88569	Nucleus
HNRNPAB	*heterogeneous nuclear ribonucleoprotein A/B*	Q99020	Nucleus
HNRNPC	*heterogeneous nuclear ribonucleoprotein C*	Q9Z204	Nucleus
HNRNPD	*heterogeneous nuclear ribonucleoprotein D*	Q60668	Nucleus
HNRNPF	*heterogeneous nuclear ribonucleoprotein F*	Q9Z2X1	Nucleus
HNRNPH1	*heterogeneous nuclear ribonucleoprotein H1*	O35737	Nucleus
HNRNPK	*heterogeneous nuclear ribonucleoprotein K*	P61979	Nucleus
HNRNPL	*heterogeneous nuclear ribonucleoprotein L*	Q8R081	Nucleus
HNRNPU	*heterogeneous nuclear ribonucleoprotein U*	Q8VEK3	Nucleus
HPRT1	*hypoxanthine phosphoribosyltransferase 1*	P00493	Cytoplasm
HSP90AA1	*heat shock protein 90 alpha family class A member 1*	P07901	Cytoplasm
HSP90AB1	*heat shock protein 90 alpha family class B member 1*	P11499	Cytoplasm
HSP90B1	*heat shock protein 90 beta family member 1*	P08113	Cytoplasm
HSPA4	*heat shock protein family A (Hsp70) member 4*	Q61316	Cytoplasm
HSPA5	*heat shock protein family A (Hsp70) member 5*	P20029	Cytoplasm
HSPA8	*heat shock protein family A (Hsp70) member 8*	P63017	Cytoplasm
HSPA13	*heat shock protein family A (Hsp70) member 13*	Q8BM72	Cytoplasm

HSPD1	*heat shock protein family D (Hsp60) member 1*	P63038	Cytoplasm
HSPE1	*heat shock protein family E (Hsp10) member 1*	Q64433	Cytoplasm
HSPG2	*heparan sulfate proteoglycan 2*	Q05793	Extracellular Space
HSPH1	*heat shock protein family H (Hsp110) member 1*	Q61699	Cytoplasm
HTRA1	*HtrA serine peptidase 1*	Q9R118	Extracellular Space
HYOU1	*hypoxia up-regulated 1*	Q9JKR6	Cytoplasm
IDH1	*isocitrate dehydrogenase (NADP(+)) 1*	O88844	Cytoplasm
IDH2	*isocitrate dehydrogenase (NADP(+)) 2*	P54071	Cytoplasm
IGFBP4	*insulin like growth factor binding protein 4*	P47879	Extracellular Space
IGFBP6	*insulin like growth factor binding protein 6*	P47880	Extracellular Space
IGHG1	*immunoglobulin heavy constant gamma 1 (G1m marker)*	P01868	Extracellular Space
ILF2	*interleukin enhancer binding factor 2*	Q9CXY6	Nucleus
IMPA1	*inositol monophosphatase 1*	O55023	Cytoplasm
IPO5	*importin 5*	Q8BKC5	Nucleus
IPO7	*importin 7*	Q9EPL8	Nucleus
IQGAP1	*IQ motif containing GTPase activating protein 1*	Q9JKF1	Cytoplasm
ITM2B	*integral membrane protein 2B*	O89051	Plasma Membrane
ITPA	*inosine triphosphatase*	Q9D892	Cytoplasm
KARS1	*lysyl-tRNA synthetase 1*	Q99MN1	Cytoplasm
KHSRP	*KH-type splicing regulatory protein*	Q3U0V1	Nucleus
KPNB1	*karyopherin subunit beta 1*	P70168	Nucleus
KRT8	*keratin 8*	P11679	Cytoplasm
KRT18	*keratin 18*	P05784	Cytoplasm
LAMA5	*laminin subunit alpha 5*	Q61001	Extracellular Space
LAMB1	*laminin subunit beta 1*	P02469	Extracellular Space
LAMC1	*laminin subunit gamma 1*	P02468	Extracellular Space
LASP1	*LIM and SH3 protein 1*	Q61792	Cytoplasm
LDHA	*lactate dehydrogenase A*	P06151	Cytoplasm
LDLR	*low density lipoprotein receptor*	P35951	Plasma Membrane
LGALS1	*galectin 1*	P16045	Extracellular Space
LGALS3	*galectin 3*	P16110	Extracellular Space
LGALS3BP	*galectin 3 binding protein*	Q07797	Plasma Membrane
LIMA1	*LIM domain and actin binding 1*	Q9ERG0	Cytoplasm
LMNA	*lamin A/C*	P48678	Nucleus
LMNB1	*lamin B1*	P14733	Nucleus
LOXL3	*lysyl oxidase like 3*	Q9Z175	Extracellular Space

LTA4H	*leukotriene A4 hydrolase*	P24527	Cytoplasm
LTBP1	*latent transforming growth factor beta binding protein 1*	Q8CG19	Extracellular Space
LTBP4	*latent transforming growth factor beta binding protein 4*	Q8K4G1	Extracellular Space
LZIC	*leucine zipper and CTNNBIP1 domain containing*	Q8K3C3	Other
MAGOH	*mago homolog, exon junction complex subunit*	P61327	Nucleus
MAN1A1	*mannosidase alpha class 1A member 1*	P45700	Cytoplasm
MAN2A1	*mannosidase alpha class 2A member 1*	P27046	Cytoplasm
MAP4	*microtubule associated protein 4*	P27546	Cytoplasm
MAP1B	*microtubule associated protein 1B*	P14873	Cytoplasm
MAPK1	*mitogen-activated protein kinase 1*	P63085	Cytoplasm
MAPK3	*mitogen-activated protein kinase 3*	Q63844	Cytoplasm
MAPRE1	*microtubule associated protein RP/EB family member 1*	Q61166	Cytoplasm
Masp1	*mannan-binding lectin serine peptidase 1*	P98064	Extracellular Space
MDH1	*malate dehydrogenase 1*	P14152	Cytoplasm
MDH2	*malate dehydrogenase 2*	P08249	Cytoplasm
ME1	*malic enzyme 1*	P06801	Cytoplasm
MEMO1	*mediator of cell motility 1*	Q91VH6	Cytoplasm
MIF	*macrophage migration inhibitory factor*	P34884	Extracellular Space
MINPP1	*multiple inositol-polyphosphate phosphatase 1*	Q9Z2L6	Cytoplasm
MMP3	*matrix metallopeptidase 3*	P28862	Extracellular Space
MSN	*moesin*	P26041	Plasma Membrane
MTAP	*methylthioadenosine phosphorylase*	Q9CQ65	Nucleus
MTPN	*myotrophin*	P62774	Nucleus
MXRA8	*matrix remodeling associated 8*	Q9DBV4	Cytoplasm
MYG1	*MYG1 exonuclease*	Q9JK81	Nucleus
MYH9	*myosin heavy chain 9*	Q8VDD5	Cytoplasm
MYH10	*myosin heavy chain 10*	Q61879	Cytoplasm
MYL6	*myosin light chain 6*	Q60605	Cytoplasm
MYL12A	*myosin light chain 12A*	Q3THE2	Cytoplasm
NACA	*nascent polypeptide associated complex subunit alpha*	Q60817	Cytoplasm
NAMPT	*nicotinamide phosphoribosyltransferase*	Q99KQ4	Extracellular Space
NAP1L1	*nucleosome assembly protein 1 like 1*	P28656	Nucleus
NARS1	*asparaginyl-tRNA synthetase 1*	Q8BP47	Cytoplasm
NASP	*nuclear autoantigenic sperm protein*	Q99MD9	Nucleus
NCL	*nucleolin*	P09405	Nucleus
Nes	*nestin*	Q6P5H2	Cytoplasm

NIBAN2	*niban apoptosis regulator 2*	Q8R1F1	Cytoplasm
NID1	*nidogen 1*	P10493	Extracellular Space
NME1	*NME/NM23 nucleoside diphosphate kinase 1*	P15532	Cytoplasm
NME2	*NME/NM23 nucleoside diphosphate kinase 2*	Q01768	Nucleus
Nolc1	*nucleolar and coiled-body phosphoprotein 1*	E9Q5C9	Nucleus
NOP56	*NOP56 ribonucleoprotein*	Q9D6Z1	Nucleus
NPEPPS	*aminopeptidase puromycin sensitive*	Q11011	Cytoplasm
NPM1	*nucleophosmin 1*	Q61937	Nucleus
NPTX1	*neuronal pentraxin 1*	Q62443	Extracellular Space
NPTXR	*neuronal pentraxin receptor*	Q99J85	Plasma Membrane
NSFL1C	*NSFL1 cofactor*	Q9CZ44	Cytoplasm
NSUN2	*NOP2/Sun RNA methyltransferase 2*	Q1HFZ0	Nucleus
NTN1	*netrin 1*	O09118	Extracellular Space
NUCB1	*nucleobindin 1*	Q02819	Cytoplasm
NUCKS1	*nuclear casein kinase and cyclin dependent kinase substrate 1*	Q80XU3	Nucleus
NUDC	*nuclear distribution C, dynein complex regulator*	O35685	Cytoplasm
NUDT21	*nudix hydrolase 21*	Q9CQF3	Nucleus
NUTF2	*nuclear transport factor 2*	P61971	Nucleus
OAF	*out at first homolog*	Q8QZR4	Cytoplasm
OLA1	*Obg like ATPase 1*	Q9CZ30	Cytoplasm
Otub1	*OTU domain, ubiquitin aldehyde binding 1*	Q7TQI3	Cytoplasm
P4HA1	*prolyl 4-hydroxylase subunit alpha 1*	Q60715	Cytoplasm
P4HB	*prolyl 4-hydroxylase subunit beta*	P09103	Cytoplasm
PA2G4	*proliferation-associated 2G4*	P50580	Nucleus
PABPC1	*poly(A) binding protein cytoplasmic 1*	P29341	Cytoplasm
PAFAH1B1	*platelet activating factor acetylhydrolase 1b regulatory subunit 1*	P63005	Cytoplasm
PAFAH1B2	*platelet activating factor acetylhydrolase 1b catalytic subunit 2*	Q61206	Cytoplasm
PAICS	*phosphoribosylaminoimidazole carboxylase and phosphoribosylaminoimidazolesuccinocarboxamide synthase*	Q9DCL9	Cytoplasm
PARK7	*Parkinsonism associated deglycase*	Q99LX0	Nucleus
PCBP1	*poly(rC) binding protein 1*	P60335	Nucleus
PCBP2	*poly(rC) binding protein 2*	Q61990	Nucleus
Pcmt1	*protein-L-isoaspartate (D-aspartate) O-methyltransferase 1*	P23506	Cytoplasm
PCNA	*proliferating cell nuclear antigen*	P17918	Nucleus
PCNP	*PEST proteolytic signal containing nuclear protein*	Q6P8I4	Nucleus
PCOLCE	*procollagen C-endopeptidase enhancer*	Q61398	Extracellular Space

PDAP1	*PDGFA associated protein 1*	Q3UHX2	Cytoplasm
PDCD5	*programmed cell death 5*	P56812	Nucleus
PDCD6IP	*programmed cell death 6 interacting protein*	Q9WU78	Cytoplasm
PDGFC	*platelet derived growth factor C*	Q8CI19	Extracellular Space
PDIA3	*protein disulfide isomerase family A member 3*	P27773	Cytoplasm
PDIA4	*protein disulfide isomerase family A member 4*	P08003	Cytoplasm
PDIA6	*protein disulfide isomerase family A member 6*	Q922R8	Cytoplasm
PDLIM1	*PDZ and LIM domain 1*	O70400	Cytoplasm
PEBP1	*phosphatidylethanolamine binding protein 1*	P70296	Cytoplasm
PFAS	*phosphoribosylformylglycinamidine synthase*	Q5SUR0	Cytoplasm
PFN1	*profilin 1*	P62962	Cytoplasm
PGAM1	*phosphoglycerate mutase 1*	Q9DBJ1	Cytoplasm
PGD	*phosphogluconate dehydrogenase*	Q9DCD0	Cytoplasm
PGK1	*phosphoglycerate kinase 1*	P09411	Cytoplasm
PGLS	*6-phosphogluconolactonase*	Q9CQ60	Cytoplasm
PGM1	*phosphoglucomutase 1*	Q9D0F9	Cytoplasm
PGM2	*phosphoglucomutase 2*	Q7TSV4	Cytoplasm
PHGDH	*phosphoglycerate dehydrogenase*	Q61753	Cytoplasm
PKM	*pyruvate kinase M1/2*	P52480	Cytoplasm
PLA2G7	*phospholipase A2 group VII*	Q60963	Extracellular Space
PLAU	*plasminogen activator, urokinase*	P06869	Extracellular Space
PLEC	*plectin*	Q9QXS1	Cytoplasm
PLIN3	*perilipin 3*	Q9DBG5	Cytoplasm
PLOD1	*procollagen-lysine,2-oxoglutarate 5-dioxygenase 1*	Q9R0E2	Cytoplasm
PLS3	*plastin 3*	Q99K51	Cytoplasm
PLTP	*phospholipid transfer protein*	P55065	Extracellular Space
PNP	*purine nucleoside phosphorylase*	P23492	Nucleus
PPA1	*inorganic pyrophosphatase 1*	Q9D819	Cytoplasm
PPIA	*peptidylprolyl isomerase A*	P17742	Cytoplasm
PPIB	*peptidylprolyl isomerase B*	P24369	Cytoplasm
PPIC	*peptidylprolyl isomerase C*	P30412	Cytoplasm
PPID	*peptidylprolyl isomerase D*	Q9CR16	Cytoplasm
PPP1CA	*protein phosphatase 1 catalytic subunit alpha*	P62137	Cytoplasm
PPP2CA	*protein phosphatase 2 catalytic subunit alpha*	P63330	Cytoplasm
PPP2R1A	*protein phosphatase 2 scaffold subunit Aalpha*	Q76MZ3	Cytoplasm
PRDX1	*peroxiredoxin 1*	P35700	Cytoplasm

PRDX2	*peroxiredoxin 2*	Q61171	Cytoplasm
PRDX5	*peroxiredoxin 5*	P99029	Cytoplasm
PRDX6	*peroxiredoxin 6*	O08709	Cytoplasm
PREP	*prolyl endopeptidase*	Q9QUR6	Cytoplasm
PRKCSH	*protein kinase C substrate 80K-H*	O08795	Cytoplasm
PRMT1	*protein arginine methyltransferase 1*	Q9JIF0	Nucleus
PROS1	*protein S*	Q08761	Extracellular Space
PRPF19	*pre-mRNA processing factor 19*	Q99KP6	Nucleus
PSAP	*prosaposin*	Q61207	Extracellular Space
PSAT1	*phosphoserine aminotransferase 1*	Q99K85	Cytoplasm
PSMA1	*proteasome 20S subunit alpha 1*	Q9R1P4	Cytoplasm
PSMA3	*proteasome 20S subunit alpha 3*	O70435	Cytoplasm
PSMA4	*proteasome 20S subunit alpha 4*	Q9R1P0	Cytoplasm
PSMA5	*proteasome 20S subunit alpha 5*	Q9Z2U1	Cytoplasm
PSMA6	*proteasome 20S subunit alpha 6*	Q9QUM9	Cytoplasm
PSMA7	*proteasome 20S subunit alpha 7*	Q9Z2U0	Cytoplasm
PSMB1	*proteasome 20S subunit beta 1*	O09061	Cytoplasm
PSMB3	*proteasome 20S subunit beta 3*	Q9R1P1	Cytoplasm
PSMB4	*proteasome 20S subunit beta 4*	P99026	Cytoplasm
PSMB5	*proteasome 20S subunit beta 5*	O55234	Cytoplasm
PSMB6	*proteasome 20S subunit beta 6*	Q60692	Nucleus
PSMC3	*proteasome 26S subunit, ATPase 3*	O88685	Nucleus
PSMC6	*proteasome 26S subunit, ATPase 6*	P62334	Nucleus
PSMD2	*proteasome 26S subunit, non-ATPase 2*	Q8VDM4	Cytoplasm
PSME1	*proteasome activator subunit 1*	P97371	Cytoplasm
PSME3	*proteasome activator subunit 3*	P61290	Cytoplasm
PTBP1	*polypyrimidine tract binding protein 1*	P17225	Nucleus
PTGES3	*prostaglandin E synthase 3*	Q9R0Q7	Cytoplasm
PTGR1	*prostaglandin reductase 1*	Q91YR9	Cytoplasm
Ptma (includes others)	*prothymosin alpha*	P26350	Nucleus
Ptms	*parathymosin*	Q9D0J8	Cytoplasm
PTPA	*protein phosphatase 2 phosphatase activator*	P58389	Cytoplasm
PUF60	*poly(U) binding splicing factor 60*	Q3UEB3	Nucleus
PXDN	*peroxidasin*	Q3UQ28	Extracellular Space
PYGB	*glycogen phosphorylase B*	Q8CI94	Cytoplasm
QSOX1	*quiescin sulfhydryl oxidase 1*	Q8BND5	Cytoplasm

RAB11B	*RAB11B, member RAS oncogene family*	P46638	Cytoplasm
RAC3	*Rac family small GTPase 3*	P60764	Cytoplasm
RACK1	*receptor for activated C kinase 1*	P68040	Cytoplasm
RAD23B	*RAD23 homolog B, nucleotide excision repair protein*	P54728	Nucleus
RALY	*RALY heterogeneous nuclear ribonucleoprotein*	Q64012	Nucleus
RAN	*RAN, member RAS oncogene family*	P62827	Nucleus
RANBP1	*RAN binding protein 1*	P34022	Nucleus
RANBP3	*RAN binding protein 3*	Q9CT10	Nucleus
RANGAP1	*Ran GTPase activating protein 1*	P46061	Nucleus
RARS1	*arginyl-tRNA synthetase 1*	Q9D0I9	Cytoplasm
RBBP4	*RB binding protein 4, chromatin remodeling factor*	Q60972	Nucleus
RBBP7	*RB binding protein 7, chromatin remodeling factor*	Q60973	Nucleus
Rbmxl1	*RNA binding motif protein, X-linked like-1*	Q91VM5	Nucleus
RCC2	*regulator of chromosome condensation 2*	Q8BK67	Nucleus
RDX	*radixin*	P26043	Cytoplasm
RHOC	*ras homolog family member C*	Q62159	Plasma Membrane
RNH1	*ribonuclease/angiogenin inhibitor 1*	Q91VI7	Cytoplasm
RNPEP	*arginyl aminopeptidase*	Q8VCT3	Cytoplasm
RPL4	*ribosomal protein L4*	Q9D8E6	Cytoplasm
RPL5	*ribosomal protein L5*	P47962	Cytoplasm
RPL6	*ribosomal protein L6*	P47911	Nucleus
RPL12	*ribosomal protein L12*	P35979	Nucleus
RPL15	*ribosomal protein L15*	Q9CZM2	Cytoplasm
RPL17	*ribosomal protein L17*	Q9CPR4	Cytoplasm
RPL22	*ribosomal protein L22*	P67984	Cytoplasm
RPL30	*ribosomal protein L30*	P62889	Cytoplasm
Rpl23a	*ribosomal protein L23A*	P62751	Nucleus
RPL7A	*ribosomal protein L7a*	P12970	Cytoplasm
RPLP0	*ribosomal protein lateral stalk subunit P0*	P14869	Cytoplasm
RPLP2	*ribosomal protein lateral stalk subunit P2*	P99027	Cytoplasm
Rplp1 (includes others)	*ribosomal protein, large, P1*	P47955	Nucleus
RPS2	*ribosomal protein S2*	P25444	Cytoplasm
RPS3	*ribosomal protein S3*	P62908	Cytoplasm
RPS6	*ribosomal protein S6*	P62754	Cytoplasm
RPS7	*ribosomal protein S7*	P62082	Cytoplasm
RPS8	*ribosomal protein S8*	P62242	Cytoplasm

RPS9	*ribosomal protein S9*	Q6ZWN5	Cytoplasm
RPS10	*ribosomal protein S10*	P63325	Cytoplasm
RPS12	*ribosomal protein S12*	P63323	Cytoplasm
RPS21	*ribosomal protein S21*	Q9CQR2	Cytoplasm
RPS25	*ribosomal protein S25*	P62852	Cytoplasm
RPS28	*ribosomal protein S28*	P62858	Cytoplasm
Rps3a1	*ribosomal protein S3A1*	P97351	Cytoplasm
RPSA	*ribosomal protein SA*	P14206	Cytoplasm
Rrbp1	*ribosome binding protein 1*	Q99PL5	Cytoplasm
RSL1D1	*ribosomal L1 domain containing 1*	Q8BVY0	Nucleus
RTN4	*reticulon 4*	Q99P72	Cytoplasm
RUVBL1	*RuvB like AAA ATPase 1*	P60122	Nucleus
S100A4	*S100 calcium binding protein A4*	P07091	Cytoplasm
S100A6	*S100 calcium binding protein A6*	P14069	Cytoplasm
SAE1	*SUMO1 activating enzyme subunit 1*	Q9R1T2	Cytoplasm
SAFB	*scaffold attachment factor B*	D3YXK2	Nucleus
SARNP	*SAP domain containing ribonucleoprotein*	Q9D1J3	Nucleus
SARS1	*seryl-tRNA synthetase 1*	P26638	Cytoplasm
SDC4	*syndecan 4*	O35988	Plasma Membrane
SDF4	*stromal cell derived factor 4*	Q61112	Cytoplasm
SEC13	*SEC13 homolog, nuclear pore and COPII coat complex component*	Q9D1M0	Cytoplasm
SEMA3E	*semaphorin 3E*	P70275	Extracellular Space
SEPHS1	*selenophosphate synthetase 1*	Q8BH69	Other
SEPTIN2	*septin 2*	P42208	Cytoplasm
SEPTIN7	*septin 7*	O55131	Cytoplasm
SEPTIN9	*septin 9*	Q80UG5	Cytoplasm
SEPTIN11	*septin 11*	Q8C1B7	Nucleus
SERBP1	*SERPINE1 mRNA binding protein 1*	Q9CY58	Cytoplasm
SERPINB6	*serpin family B member 6*	Q60854	Cytoplasm
SERPINF1	*serpin family F member 1*	P97298	Extracellular Space
SERPINH1	*serpin family H member 1*	P19324	Extracellular Space
SET	*SET nuclear proto-oncogene*	Q9EQU5	Nucleus
Sf1	*splicing factor 1*	Q64213	Nucleus
SF3A1	*splicing factor 3a subunit 1*	Q8K4Z5	Nucleus
SF3B1	*splicing factor 3b subunit 1*	Q99NB9	Nucleus
SF3B3	*splicing factor 3b subunit 3*	Q921M3	Nucleus

SFPQ	*splicing factor proline and glutamine rich*	Q8VIJ6	Nucleus
SH3BGRL3	*SH3 domain binding glutamate rich protein like 3*	Q91VW3	Nucleus
SH3BGRL	*SH3 domain binding glutamate rich protein like*	Q9JJU8	Cytoplasm
SH3KBP1	*SH3 domain containing kinase binding protein 1*	Q8R550	Cytoplasm
SNRNP70	*small nuclear ribonucleoprotein U1 subunit 70*	Q62376	Nucleus
SNRNP200	*small nuclear ribonucleoprotein U5 subunit 200*	Q6P4T2	Nucleus
SNRPB	*small nuclear ribonucleoprotein polypeptides B and B1*	P27048	Nucleus
SNRPD2	*small nuclear ribonucleoprotein D2 polypeptide*	P62317	Nucleus
SNRPD3	*small nuclear ribonucleoprotein D3 polypeptide*	P62320	Nucleus
Snrpe	*small nuclear ribonucleoprotein E*	P62305	Nucleus
SNW1	*SNW domain containing 1*	Q9CSN1	Nucleus
SNX9	*sorting nexin 9*	Q91VH2	Cytoplasm
SOD1	*superoxide dismutase 1*	P08228	Cytoplasm
SPARC	*secreted protein acidic and cysteine rich*	P07214	Extracellular Space
SPP1	*secreted phosphoprotein 1*	P10923	Extracellular Space
SPTAN1	*spectrin alpha, non-erythrocytic 1*	P16546	Plasma Membrane
SPTBN1	*spectrin beta, non-erythrocytic 1*	Q62261	Plasma Membrane
SRM	*spermidine synthase*	Q64674	Cytoplasm
Srrm2	*serine/arginine repetitive matrix 2*	Q8BTI8	Nucleus
SRSF1	*serine and arginine rich splicing factor 1*	Q6PDM2	Nucleus
SRSF2	*serine and arginine rich splicing factor 2*	Q62093	Nucleus
SSB	*small RNA binding exonuclease protection factor La*	P32067	Nucleus
SSRP1	*structure specific recognition protein 1*	Q08943	Nucleus
ST13	*ST13 Hsp70 interacting protein*	Q99L47	Cytoplasm
STC1	*stanniocalcin 1*	O55183	Extracellular Space
STIP1	*stress induced phosphoprotein 1*	Q60864	Cytoplasm
STRAP	*serine/threonine kinase receptor associated protein*	Q9Z1Z2	Plasma Membrane
SUB1	*SUB1 regulator of transcription*	P11031	Nucleus
SUPT16H	*SPT16 homolog, facilitates chromatin remodeling subunit*	Q920B9	Nucleus
SYNCRIP	*synaptotagmin binding cytoplasmic RNA interacting protein*	Q7TMK9	Nucleus
TAGLN2	*transgelin 2*	Q9WVA4	Cytoplasm
TALDO1	*transaldolase 1*	Q93092	Cytoplasm
TBCA	*tubulin folding cofactor A*	P48428	Cytoplasm
TCEA1	*transcription elongation factor A1*	P10711	Nucleus
TCOF1	*treacle ribosome biogenesis factor 1*	O08784	Nucleus
TCP1	*t-complex 1*	P11983	Cytoplasm

TFPI	*tissue factor pathway inhibitor*	O54819	Extracellular Space
TGFBR3	*transforming growth factor beta receptor 3*	O88393	Plasma Membrane
THBS1	*thrombospondin 1*	P35441	Extracellular Space
THBS2	*thrombospondin 2*	Q03350	Extracellular Space
THOP1	*thimet oligopeptidase 1*	Q8C1A5	Cytoplasm
THRAP3	*thyroid hormone receptor associated protein 3*	Q569Z6	Nucleus
TIMM13	*translocase of inner mitochondrial membrane 13*	P62075	Cytoplasm
TIMP2	*TIMP metallopeptidase inhibitor 2*	P25785	Extracellular Space
TKT	*transketolase*	P40142	Cytoplasm
TLN1	*talin 1*	P26039	Plasma Membrane
TMPO	*thymopoietin*	Q61029	Nucleus
Tmsb4x (includes others)	*thymosin, beta 4, X chromosome*	Q6ZWY8	Cytoplasm
TPD52L2	*TPD52 like 2*	Q9CYZ2	Cytoplasm
TPI1	*triosephosphate isomerase 1*	P17751	Cytoplasm
Tpm1	*tropomyosin 1, alpha*	P58771	Plasma Membrane
TPM3	*tropomyosin 3*	P21107	Cytoplasm
Tpm4	*tropomyosin 4*	Q6IRU2	Cytoplasm
TPT1	*tumor protein, translationally-controlled 1*	P63028	Cytoplasm
TRIM28	*tripartite motif containing 28*	Q62318	Nucleus
TSN	*translin*	Q62348	Nucleus
TUBA1A	*tubulin alpha 1a*	P68369	Cytoplasm
TUBB	*tubulin beta class I*	P99024	Cytoplasm
TUBB4B	*tubulin beta 4B class IVb*	P68372	Cytoplasm
TXN	*thioredoxin*	P10639	Cytoplasm
TXNDC17	*thioredoxin domain containing 17*	Q9CQM5	Cytoplasm
TXNL1	*thioredoxin like 1*	Q8CDN6	Cytoplasm
TXNRD1	*thioredoxin reductase 1*	Q9JMH6	Cytoplasm
U2AF2	*U2 small nuclear RNA auxiliary factor 2*	P26369	Nucleus
UAP1L1	*UDP-N-acetylglucosamine pyrophosphorylase 1 like 1*	Q3TW96	Other
UBA1	*ubiquitin like modifier activating enzyme 1*	Q02053	Cytoplasm
Ubb	*ubiquitin B*	P0CG49	Cytoplasm
UBE2I	*ubiquitin conjugating enzyme E2 I*	P63280	Nucleus
UBE2K	*ubiquitin conjugating enzyme E2 K*	P61087	Cytoplasm
UBE2L3	*ubiquitin conjugating enzyme E2 L3*	P68037	Nucleus
UBE2M	*ubiquitin conjugating enzyme E2 M*	P61082	Cytoplasm
UBE2N	*ubiquitin conjugating enzyme E2 N*	P61089	Cytoplasm

UBE2V2	*ubiquitin conjugating enzyme E2 V2*	Q9D2M8	Cytoplasm
UCHL3	*ubiquitin C-terminal hydrolase L3*	Q9JKB1	Cytoplasm
USP5	*ubiquitin specific peptidase 5*	P56399	Cytoplasm
USP14	*ubiquitin specific peptidase 14*	Q9JMA1	Cytoplasm
VARS1	*valyl-tRNA synthetase 1*	Q9Z1Q9	Cytoplasm
VASN	*vasorin*	Q9CZT5	Plasma Membrane
VAT1	*vesicle amine transport 1*	Q62465	Plasma Membrane
VCAN	*versican*	Q62059	Extracellular Space
VCL	*vinculin*	Q64727	Plasma Membrane
VCP	*valosin containing protein*	Q01853	Cytoplasm
VIM	*vimentin*	P20152	Cytoplasm
WDR1	*WD repeat domain 1*	O88342	Extracellular Space
XPO1	*exportin 1*	Q6P5F9	Nucleus
YBX1	*Y-box binding protein 1*	P62960	Nucleus
YWHAB	*tyrosine 3-monooxygenase/tryptophan 5-monooxygenase activation protein beta*	Q9CQV8	Cytoplasm
YWHAE	*tyrosine 3-monooxygenase/tryptophan 5-monooxygenase activation protein epsilon*	P62259	Cytoplasm
YWHAG	*tyrosine 3-monooxygenase/tryptophan 5-monooxygenase activation protein gamma*	P61982	Cytoplasm
YWHAH	*tyrosine 3-monooxygenase/tryptophan 5-monooxygenase activation protein eta*	P68510	Cytoplasm
YWHAQ	*tyrosine 3-monooxygenase/tryptophan 5-monooxygenase activation protein theta*	P68254	Cytoplasm
YWHAZ	*tyrosine 3-monooxygenase/tryptophan 5-monooxygenase activation protein zeta*	P63101	Cytoplasm
ZYX	*zyxin*	Q62523	Plasma Membrane

Table S4. List of A549 secretome profiling.

Entrez Gene Name	GenPept/UniProt/Swiss-Prot Accession	Location	Type(s)
ATP citrate lyase	P53396	Cytoplasm	enzyme
actin beta	P60709	Cytoplasm	other
actin gamma 1	P63261	Cytoplasm	other
actinin alpha 1	P12814	Cytoplasm	transcription regulator
actinin alpha 4	O43707	Cytoplasm	transcription regulator
adhesion G protein-coupled receptor G6	Q86SQ4	Plasma Membrane	G-protein coupled receptor
alpha fetoprotein	P02771	Extracellular Space	transporter
agrin	O00468	Plasma Membrane	other

alpha 2-HS glycoprotein	P02765	Extracellular Space	other
adenylate kinase 2	P54819	Cytoplasm	kinase
aldo-keto reductase family 1 member B	P15121	Cytoplasm	enzyme
aldo-keto reductase family 1 member B10	O60218	Cytoplasm	enzyme
aldo-keto reductase family 1 member C3	P42330	Cytoplasm	enzyme
aldo-keto reductase family 1 member C2	P52895	Cytoplasm	enzyme
albumin	P02768	Extracellular Space	transporter
activated leukocyte cell adhesion molecule	Q13740	Plasma Membrane	other
aldehyde dehydrogenase 1 family member A1	P00352	Cytoplasm	enzyme
aldolase, fructose-bisphosphate A	P04075	Cytoplasm	enzyme
angiogenin	P03950	Extracellular Space	enzyme
apurinic/apyrimidinic endodeoxyribonuclease 1	P27695	Nucleus	enzyme
amyloid beta precursor protein	P05067	Plasma Membrane	other
AXL receptor tyrosine kinase	P30530	Plasma Membrane	kinase
beta-2-microglobulin	P61769	Plasma Membrane	transmembrane receptor
beta-1,4-galactosyltransferase 1	P15291	Cytoplasm	enzyme
beta-1,4-glucuronyltransferase 1	O43505	Cytoplasm	enzyme
brain abundant membrane attached signal protein 1	P80723	Nucleus	transcription regulator
basal cell adhesion molecule (Lutheran blood group)	P50895	Plasma Membrane	transmembrane receptor
complement C3	P01024	Extracellular Space	peptidase
complement C5	P01031	Extracellular Space	cytokine
complement C1r	P00736	Extracellular Space	peptidase
complement C1s	P09871	Extracellular Space	peptidase
calreticulin	P27797	Cytoplasm	transcription regulator
capping actin protein of muscle Z-line subunit beta	P47756	Cytoplasm	other
C-C motif chemokine ligand 2	P13500	Extracellular Space	cytokine
cellular communication network factor 1	O00622	Extracellular Space	other
cellular communication network factor 2	P29279	Extracellular Space	growth factor
CD44 molecule (Indian blood group)	P16070	Plasma Membrane	other
cadherin 2	P19022	Plasma Membrane	other
complement factor B	P00751	Extracellular Space	peptidase
complement factor D	P00746	Extracellular Space	peptidase
complement factor H	P08603	Extracellular Space	other
complement factor I	P05156	Extracellular Space	peptidase
cofilin 1	P23528	Nucleus	other
chloride intracellular channel 1	O00299	Nucleus	ion channel

calsyntenin 1	O94985	Plasma Membrane	other
clusterin	P10909	Cytoplasm	other
cytidine/uridine monophosphate kinase 1	P30085	Nucleus	kinase
contactin 1	Q12860	Plasma Membrane	enzyme
cochlin	O43405	Extracellular Space	other
collagen type XVIII alpha 1 chain	P39060	Extracellular Space	other
collagen type V alpha 1 chain	P20908	Extracellular Space	other
collagen type V alpha 2 chain	P05997	Extracellular Space	other
collagen type VI alpha 1 chain	P12109	Extracellular Space	other
collagen type VII alpha 1 chain	Q02388	Extracellular Space	other
ceruloplasmin	P00450	Extracellular Space	enzyme
cysteine rich transmembrane BMP regulator 1	Q9NZV1	Extracellular Space	kinase
colony stimulating factor 1	P09603	Extracellular Space	cytokine
cystatin C	P01034	Extracellular Space	other
cathepsin B	P07858	Cytoplasm	peptidase
cathepsin D	P07339	Cytoplasm	peptidase
cathepsin L	P07711	Cytoplasm	peptidase
cathepsin Z	Q9UBR2	Cytoplasm	peptidase
C-X-C motif chemokine ligand 1	P09341	Extracellular Space	cytokine
C-X-C motif chemokine ligand 3	P19876	Extracellular Space	cytokine
C-X-C motif chemokine ligand 5	P42830	Extracellular Space	cytokine
C-X-C motif chemokine ligand 8	P10145	Extracellular Space	cytokine
dystroglycan 1	Q14118	Plasma Membrane	transmembrane receptor
dickkopf WNT signaling pathway inhibitor 1	O94907	Extracellular Space	growth factor
EGF containing fibulin extracellular matrix protein 1	Q12805	Extracellular Space	enzyme
eukaryotic translation initiation factor 4A1	P60842	Cytoplasm	translation regulator
eukaryotic translation initiation factor 4B	P23588	Cytoplasm	translation regulator
eukaryotic translation initiation factor 5A	P63241	Cytoplasm	translation regulator
enolase 1	P06733	Cytoplasm	enzyme
family with sequence similarity 3 member C	Q92520	Extracellular Space	cytokine
fibulin 1	P23142	Extracellular Space	other
filamin A	P21333	Cytoplasm	other
filamin B	O75369	Cytoplasm	other
fibronectin 1	P02751	Extracellular Space	enzyme
fascin actin-bundling protein 1	Q16658	Cytoplasm	other
follistatin like 1	Q12841	Extracellular Space	other

follistatin like 3	O95633	Extracellular Space	other
polypeptide N-acetylgalactosaminyltransferase 2	Q10471	Cytoplasm	enzyme
GDP dissociation inhibitor 2	P50395	Cytoplasm	other
glutaredoxin 3	O76003	Cytoplasm	enzyme
glucosamine (N-acetyl)-6-sulfatase	P15586	Cytoplasm	enzyme
golgi membrane protein 1	Q8NBJ4	Cytoplasm	other
glypican 1	P35052	Plasma Membrane	transmembrane receptor
glucose-6-phosphate isomerase	P06744	Extracellular Space	enzyme
granulin precursor	P28799	Extracellular Space	growth factor
glutathione S-transferase pi 1	P09211	Cytoplasm	enzyme
heparin binding growth factor	P51858	Extracellular Space	growth factor
high mobility group box 1	P09429	Nucleus	transcription regulator
heat shock protein 90 alpha family class A member 1	P07900	Cytoplasm	enzyme
heat shock protein family A (Hsp70) member 5	P11021	Cytoplasm	enzyme
heat shock protein family A (Hsp70) member 8	P11142	Cytoplasm	enzyme
heat shock protein family A (Hsp70) member 9	P38646	Cytoplasm	other
heat shock protein family A (Hsp70) member 1A	P0DMV8	Cytoplasm	enzyme
heat shock protein family E (Hsp10) member 1	P61604	Cytoplasm	enzyme
heparan sulfate proteoglycan 2	P98160	Extracellular Space	enzyme
insulin like growth factor binding protein 1	P08833	Extracellular Space	other
insulin like growth factor binding protein 3	P17936	Extracellular Space	other
insulin like growth factor binding protein 4	P22692	Extracellular Space	other
insulin like growth factor binding protein 6	P24592	Extracellular Space	other
insulin like growth factor binding protein 7	Q16270	Extracellular Space	transporter
jagged canonical Notch ligand 1	P78504	Extracellular Space	growth factor
keratin 8	P05787	Cytoplasm	other
keratin 18	P05783	Cytoplasm	other
kynureninase	Q16719	Cytoplasm	enzyme
laminin subunit alpha 5	O15230	Extracellular Space	other
laminin subunit beta 1	P07942	Extracellular Space	other
laminin subunit gamma 1	P11047	Extracellular Space	other
lactate dehydrogenase A	P00338	Cytoplasm	enzyme
lactate dehydrogenase B	P07195	Cytoplasm	enzyme
low density lipoprotein receptor	P01130	Plasma Membrane	transporter
galectin 3 binding protein	Q08380	Plasma Membrane	transmembrane receptor
legumain	Q99538	Cytoplasm	peptidase

latent transforming growth factor beta binding protein 3	Q9NS15	Extracellular Space	other
latent transforming growth factor beta binding protein 4	Q8N2S1	Extracellular Space	growth factor
malate dehydrogenase 1	P40925	Cytoplasm	enzyme
malate dehydrogenase 2	P40926	Cytoplasm	enzyme
matrix metallopeptidase 2	P08253	Extracellular Space	peptidase
moesin	P26038	Plasma Membrane	other
mucin 5B, oligomeric mucus/gel-forming	Q9HC84	Extracellular Space	peptidase
nuclear autoantigenic sperm protein	P49321	Nucleus	other
nucleolin	P19338	Nucleus	other
neogenin 1	Q92859	Plasma Membrane	transcription regulator
NME/NM23 nucleoside diphosphate kinase 1	P15531	Cytoplasm	kinase
NME/NM23 nucleoside diphosphate kinase 2	P22392	Nucleus	kinase
nucleolar and coiled-body phosphoprotein 1	Q14978	Nucleus	transcription regulator
NPC intracellular cholesterol transporter 2	P61916	Extracellular Space	transporter
aminopeptidase puromycin sensitive	P55786	Cytoplasm	peptidase
nucleophosmin 1	P06748	Nucleus	transcription regulator
neuronal cell adhesion molecule	Q92823	Plasma Membrane	other
neuregulin 1	Q02297	Plasma Membrane	growth factor
neuropilin 2	O60462	Plasma Membrane	kinase
nucleobindin 1	Q02818	Cytoplasm	other
nuclear casein kinase and cyclin dependent kinase substrate 1	Q9H1E3	Nucleus	kinase
proliferation-associated 2G4	Q9UQ80	Nucleus	transcription regulator
pappalysin 1	Q13219	Extracellular Space	peptidase
Parkinsonism associated deglycase	Q99497	Nucleus	enzyme
proliferating cell nuclear antigen	P12004	Nucleus	enzyme
PEST proteolytic signal containing nuclear protein	Q8WW12	Nucleus	other
proprotein convertase subtilisin/kexin type 9	Q8NBP7	Extracellular Space	peptidase
phosphatidylethanolamine binding protein 1	P30086	Cytoplasm	other
peptidase D	P12955	Cytoplasm	peptidase
profilin 1	P07737	Cytoplasm	other
phosphoglycerate mutase 1	P18669	Cytoplasm	phosphatase
phosphogluconate dehydrogenase	P52209	Cytoplasm	enzyme
phosphoglycerate kinase 1	P00558	Cytoplasm	kinase
pyruvate kinase M1/2	P14618	Cytoplasm	kinase
plastin 3	P13797	Cytoplasm	other
pyridoxamine 5′-phosphate oxidase	Q9NVS9	Cytoplasm	enzyme

peptidylprolyl isomerase A	P62937	Cytoplasm	enzyme
peptidylprolyl isomerase B	P23284	Cytoplasm	enzyme
peroxiredoxin 1	Q06830	Cytoplasm	enzyme
protein S	P07225	Extracellular Space	other
prosaposin	P07602	Extracellular Space	enzyme
proteasome subunit alpha 1	P25786	Cytoplasm	peptidase
proteasome subunit alpha 5	P28066	Cytoplasm	peptidase
prostaglandin reductase 1	Q14914	Cytoplasm	enzyme
prothymosin alpha	P06454	Nucleus	other
parathymosin	P20962	Nucleus	other
protein tyrosine phosphatase, receptor type F	P10586	Plasma Membrane	phosphatase
poliovirus receptor	P15151	Plasma Membrane	other
quiescin sulfhydryl oxidase 1	O00391	Cytoplasm	enzyme
retinoic acid receptor responder 1	P49788	Plasma Membrane	other
ribonuclease A family member 4	P34096	Extracellular Space	enzyme
ribosomal protein lateral stalk subunit P1	P05386	Cytoplasm	other
syndecan 4	P31431	Plasma Membrane	other
shisa family member 5	Q8N114	Nucleus	other
solute carrier family 3 member 2	P08195	Plasma Membrane	transporter
SPARC related modular calcium binding 1	Q9H4F8	Extracellular Space	other
superoxide dismutase 1	P00441	Cytoplasm	enzyme
SPARC (osteonectin), cwcv and kazal like domains proteoglycan 1	Q08629	Extracellular Space	other
secreted phosphoprotein 1	P10451	Extracellular Space	cytokine
serglycin	P10124	Cytoplasm	other
stanniocalcin 1	P52823	Extracellular Space	kinase
transgelin 2	P37802	Cytoplasm	other
transaldolase 1	P37837	Cytoplasm	enzyme
tubulin folding cofactor A	O75347	Cytoplasm	other
tissue factor pathway inhibitor	P10646	Extracellular Space	other
transforming growth factor beta 2	P61812	Extracellular Space	growth factor
transforming growth factor beta induced	Q15582	Extracellular Space	other
thrombospondin 1	P07996	Extracellular Space	other
TIMP metallopeptidase inhibitor 1	P01033	Extracellular Space	cytokine
TIMP metallopeptidase inhibitor 2	P16035	Extracellular Space	other
transketolase	P29401	Cytoplasm	enzyme
transmembrane protein 132A	Q24JP5	Cytoplasm	other

thymosin beta 4 X-linked	P63313	Cytoplasm	other
TNF receptor superfamily member 12A	Q9NP84	Plasma Membrane	transmembrane receptor
TNF receptor superfamily member 1A	P19438	Plasma Membrane	transmembrane receptor
triosephosphate isomerase 1	P60174	Cytoplasm	enzyme
tropomyosin 4	P67936	Cytoplasm	other
tripeptidyl peptidase 1	O14773	Cytoplasm	peptidase
thioredoxin reductase 1	Q16881	Cytoplasm	enzyme
ubiquitin C	P0CG48	Cytoplasm	enzyme
versican	P13611	Extracellular Space	other
valosin containing protein	P55072	Cytoplasm	enzyme
very low density lipoprotein receptor	P98155	Plasma Membrane	transporter
tyrosine 3-monooxygenase/tryptophan 5-monooxygenase activation protein epsilon	P62258	Cytoplasm	other
tyrosine 3-monooxygenase/tryptophan 5-monooxygenase activation protein gamma	P61981	Cytoplasm	other
tyrosine 3-monooxygenase/tryptophan 5-monooxygenase activation protein theta	P27348	Cytoplasm	other
tyrosine 3-monooxygenase/tryptophan 5-monooxygenase activation protein zeta	P63104	Cytoplasm	enzyme

Table S5. List of materials used in the study.

Ragent or Resource	Source	Identifier	Working Status
Antibodies	Company		
Anti-MCP1	Abclonal	Cat# A7277	1/200 for IHC, 1/500 for Western
Anti-IL-6	Abclonal	Cat# A02861	1/100 for IHC, 1/500 for Western
Anti-TGF-β	Arigo	Cat# ARG1002	1/200 for IHC, 1/500 for Western
Anti-β-Actin	Abcam	Cat# SI-A5441-.2 ml	1/1,000 for Western
anti-**α**-actinin	Santa Cruz		
anti-synatopodin	Santa Cruz		
Cell lines			
NRK-52E	ATCC	ATCC® CCL-1571	DMEM + 10%FBS
LLC1	ATCC	ATCC® CCL-1642	DMEM + 10%FBS
A549	ATCC	ATCC® CCL-185	RPMI + 10%FBS
Chemicals, Enzymes and Materials			
Periodic Acid-Schiff kit	Sigma	Cat# 395B-1KT	
Immunohistochemical stain kit	ThermoFisher	Cat# 32020	
RIPA buffer	ThermoFisher	Cat# 89900	
Protease inhibitor cocktail	Biotools	Cat# TAAR-BBI2	
Phosphatase inhibitor cocktail	Biotools	Cat# TAAR-BBI3	
Pierce BCA protein assy kit	ThermoFisher	Cat# 23225	
PVDF membrane	Merck	Cat# IEVH00005	
Protein standard	ThermoFisher	Cat# 26616	
Fetal bovine serum	Gibco	Cat# 10437028	
Dulbecco's Modified Eagle Medium	Gibco	Cat# 12100061	
Penicillium/Stretomycin	Gibco	Cat# 15140122	
RPMI 1640	Gibco	Cat# 21875034	
Glutamine	ThermoFisher	Cat# 25030081	
Others			
Leica DM16000 B microscope	Leica	N/A	
Leica SM2125 Microtome	Leica	N/A	
Panoramic MIDI digital scanner	3DHISTECH	N/A	
UVP Bioimaging system	UVP	N/A	
Roche cobas 600c 501 analyzers	Roche	N/A	
Roche Cobas b221	Roche	N/A	
Prism 6	GraphPad	N/A	

References

1. Chung, A.C.; Lan, H.Y. Chemokines in renal injury. *J. Am. Soc. Nephrol* **2011**, *22*, 802–809, doi:10.1681/ASN.2010050510.
2. Nakaya, I.; Wada, T.; Furuichi, K.; Sakai, N.; Kitagawa, K.; Yokoyama, H.; Ishida, Y.; Kondo, T.; Sugaya, T.; Kawachi, H., et al. Blockade of IP-10/CXCR3 promotes progressive renal fibrosis. *Nephron Exp. Nephrol* **2007**, *107*, 12–21, doi:10.1159/000106505.
3. Najjar, Y.G.; Rayman, P.; Jia, X.; Pavicic, P.G., Jr.; Rini, B.I.; Tannenbaum, C.; Ko, J.; Haywood, S.; Cohen, P.; Hamilton, T., et al. Myeloid-Derived Suppressor Cell Subset Accumulation in Renal Cell Carcinoma

Parenchyma Is Associated with Intratumoral Expression of IL1beta, IL8, CXCL5, and Mip-1alpha. *Clin. Cancer Res.* **2017**, *23*, 2346–2355, doi:10.1158/1078-0432.CCR-15-1823.

4. Bedke, J.; Nelson, P.J.; Kiss, E.; Muenchmeier, N.; Rek, A.; Behnes, C.L.; Gretz, N.; Kungl, A.J.; Grone, H.J. A novel CXCL8 protein-based antagonist in acute experimental renal allograft damage. *Mol. Immunol* **2010**, *47*, 1047–1057, doi:10.1016/j.molimm.2009.11.012.
5. Cassini, M.F.; Kakade, V.R.; Kurtz, E.; Sulkowski, P.; Glazer, P.; Torres, R.; Somlo, S.; Cantley, L.G. Mcp1 Promotes Macrophage-Dependent Cyst Expansion in Autosomal Dominant Polycystic Kidney Disease. *J. Am. Soc. Nephrol* **2018**, *29*, 2471–2481, doi:10.1681/ASN.2018050518.
6. Kaleta, B. The role of osteopontin in kidney diseases. *Inflamm Res.* **2019**, *68*, 93–102, doi:10.1007/s00011-018-1200-5.
7. Yu, L.; Border, W.A.; Huang, Y.; Noble, N.A. TGF-beta isoforms in renal fibrogenesis. *Kidney Int* **2003**, *64*, 844–856, doi:10.1046/j.1523-1755.2003.00162.x.
8. Gomez, I.G.; Roach, A.M.; Nakagawa, N.; Amatucci, A.; Johnson, B.G.; Dunn, K.; Kelly, M.C.; Karaca, G.; Zheng, T.S.; Szak, S., et al. TWEAK-Fn14 Signaling Activates Myofibroblasts to Drive Progression of Fibrotic Kidney Disease. *J. Am. Soc. Nephrol* **2016**, *27*, 3639–3652, doi:10.1681/ASN.2015111227.
9. Huang, B.; Cheng, Y.; Usa, K.; Liu, Y.; Baker, M.A.; Mattson, D.L.; He, Y.; Wang, N.; Liang, M. Renal Tumor Necrosis Factor alpha Contributes to Hypertension in Dahl Salt-Sensitive Rats. *Sci Rep.* **2016**, *6*, 21960, doi:10.1038/srep21960.

Publisher's Note: MDPI stays neutral with regard to jurisdictional claims in published maps and institutional affiliations.

© 2020 by the authors. Licensee MDPI, Basel, Switzerland. This article is an open access article distributed under the terms and conditions of the Creative Commons Attribution (CC BY) license (http://creativecommons.org/licenses/by/4.0/).
